# Live-attenuated vaccine sCPD9 elicits superior mucosal and systemic immunity to SARS-CoV-2 variants in hamsters

**DOI:** 10.1038/s41564-023-01352-8

**Published:** 2023-04-03

**Authors:** Geraldine Nouailles, Julia M. Adler, Peter Pennitz, Stefan Peidli, Luiz Gustavo Teixeira Alves, Morris Baumgardt, Judith Bushe, Anne Voss, Alina Langenhagen, Christine Langner, Ricardo Martin Vidal, Fabian Pott, Julia Kazmierski, Aileen Ebenig, Mona V. Lange, Michael D. Mühlebach, Cengiz Goekeri, Szandor Simmons, Na Xing, Azza Abdelgawad, Susanne Herwig, Günter Cichon, Daniela Niemeyer, Christian Drosten, Christine Goffinet, Markus Landthaler, Nils Blüthgen, Haibo Wu, Martin Witzenrath, Achim D. Gruber, Samantha D. Praktiknjo, Nikolaus Osterrieder, Emanuel Wyler, Dusan Kunec, Jakob Trimpert

**Affiliations:** 1 Department of Infectious Diseases, Respiratory Medicine and Critical Care, Charité – Universitätsmedizin Berlin, corporate member of Freie Universität Berlin and Humboldt-Universität zu Berlin, Berlin, Germany; 2 Institut für Virologie, Freie Universität Berlin, Berlin, Germany; 3 Institute of Pathology Charité - Universitätsmedizin Berlin, corporate member of Freie Universität Berlin and Humboldt-Universität zu Berlin, and Institute for Biology, IRI Life Sciences, Humboldt-Universität zu Berlin, Berlin, Germany; 4 Berlin Institute for Medical Systems Biology (BIMSB), Max Delbrück Center for Molecular Medicine in the Helmholtz Association (MDC), Berlin, Germany; 5 Institut für Tierpathologie, Freie Universität Berlin, Berlin, Germany; 6 Institute of Virology, Charité - Universitätsmedizin Berlin, corporate member of Freie Universität Berlin and Humboldt-Universität zu Berlin, Berlin, Germany; 7 Berlin Institute of Health (BIH), Berlin, Germany; 8 Product Testing of IVMPs, Division of Veterinary Medicines, Paul-Ehrlich-Institut, Langen, Germany; 9 German Center for Infection Research (DZIF), partner site Gießen-Marburg-Langen, Giessen, Germany; 10 Faculty of Medicine, Cyprus International University, Nicosia, Cyprus; 11 Institute of Physiology, Charité - Universitätsmedizin Berlin, corporate member of Freie Universität Berlin and Humboldt-Universität zu Berlin, Berlin, Germany; 12 Department of Gynecology, Charité - Universitätsmedizin Berlin, corporate member of Freie Universität Berlin and Humboldt-Universität zu Berlin, Berlin, Germany; 13 German Center for Infection Research (DZIF), partner site Charité, Berlin, Germany; 14 Berlin Institute for Medical Systems Biology (BIMSB) Max Delbrück Center for Molecular Medicine in the Helmholtz Association (MDC), and Institute for Biology, Humboldt-Universität zu Berlin, Berlin, Germany; 15 School of Life Sciences, Chongqing University, Chongqing, China; 16 Berlin Institute of Health at Charité, Universitätsmedizin Berlin, Berlin, Germany; 17 Department of Infectious Diseases and Public Health, Jockey Club College of Veterinary Medicine and Life Sciences, City University of Hong Kong, Kowloon, Hong Kong China

**Keywords:** Live attenuated vaccines, SARS-CoV-2, Mucosal immunology

## Abstract

Vaccines play a critical role in combating the COVID-19 pandemic. Future control of the pandemic requires improved vaccines with high efficacy against newly emerging SARS-CoV-2 variants and the ability to reduce virus transmission. Here we compare immune responses and preclinical efficacy of the mRNA vaccine BNT162b2, the adenovirus-vectored spike vaccine Ad2-spike and the live-attenuated virus vaccine candidate sCPD9 in Syrian hamsters, using both homogeneous and heterologous vaccination regimens. Comparative vaccine efficacy was assessed by employing readouts from virus titrations to single-cell RNA sequencing. Our results show that sCPD9 vaccination elicited the most robust immunity, including rapid viral clearance, reduced tissue damage, fast differentiation of pre-plasmablasts, strong systemic and mucosal humoral responses, and rapid recall of memory T cells from lung tissue after challenge with heterologous SARS-CoV-2. Overall, our results demonstrate that live-attenuated vaccines offer advantages over currently available COVID-19 vaccines.

## Abstract (web summary)

Comparison of mucosal and systemic immunity after vaccination with the live-attenuated vaccine sCPD9, mRNA vaccine BNT162b2 or an adenovirus-vectored vaccine following SARS-CoV-2 challenge in hamsters.

## Main

As of 2023, 13 COVID-19 vaccines have met the standards for emergency use listing (EUL) by the WHO^1^. Authorized vaccines include inactivated virus and subunit vaccines, adenoviral-vectored spike and nucleoside-modified mRNA vaccines^2^. While available vaccines provide long-lasting protection from severe illness, waning of immunity is evident, particularly following the emergence and spread of omicron variants^3,4^.

Optimal COVID-19 vaccines protect from severe disease, span a broad spectrum of virus variants and prevent or limit SARS-CoV-2 transmission. Live-attenuated vaccines (LAV), which have been successfully used against virus infections such as measles, mumps and rubella (MMR)^5^, offer advantages over other types of vaccines. They do not require adjuvants^6^ and can be administered locally, for example intranasally, as in the case of influenza LAVs^7^. Composed of replication-competent viruses, intranasal LAVs mimic the natural course of infection and antigen production, which distinguishes them from locally administered, replication-incompetent vector- or antigen-based vaccines^8^. In contrast to empirically generated vaccines used in the past, modern LAV design utilizes molecular tools to limit virus replication and virulence while maintaining immunogenicity and antigenic integrity^9^. One recent strategy employed in the rational design of LAVs is codon pair deoptimization (CPD), which is suitable for both DNA^10^ and RNA viruses^11^, including SARS-CoV-2^12^.

Current COVID-19 vaccines are administered intramuscularly and efficiently induce systemic immunity, including high titre of neutralizing antibodies, central and effector memory T cells^13^, nasal-resident CD8^+^ T cells^14^, germinal centre B cells^15^ and long-lived plasma cells^16^. However, this route is less effective in inducing durable mucosal IgA and IgG responses^17,18^ and pulmonary tissue-resident memory cell responses^19^. Mucosal antibodies at the site of virus entry play crucial roles in limiting infectivity and transmission^20^. Accordingly, tissue-resident memory cells undergo faster recall responses due to local positioning and allow earlier cognate antigen recognition^21^. Hence, vaccines administered via respiratory routes are expected to provide robust local mucosal immunity against targeted pathogens^22^. Here we compare different vaccines and vaccine regimens, evaluating systemic and mucosal immunity conferred by each vaccine.

## Results

**Fig. 1:**
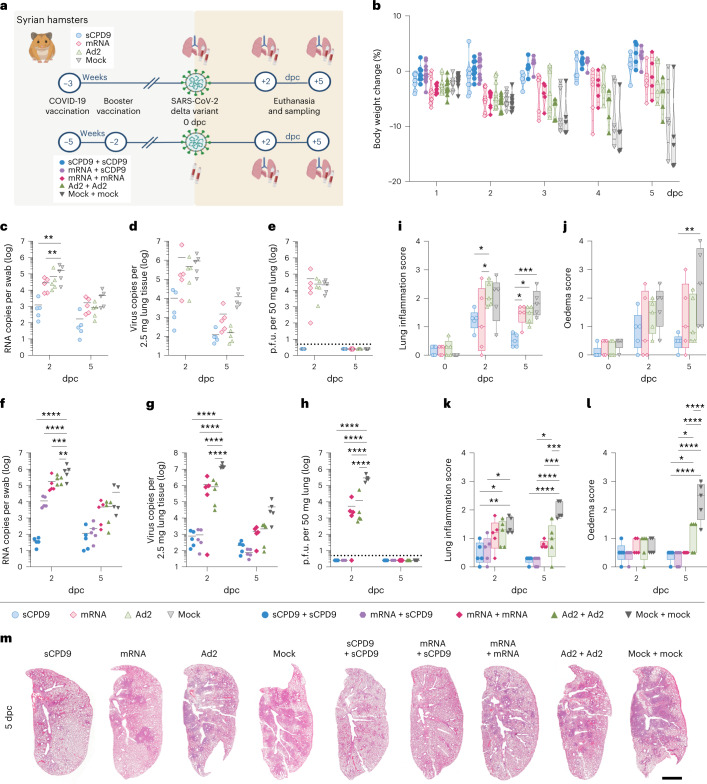
Disease severity following SARS-CoV-2 infection in vaccinated and non-vaccinated hamsters. **a**, Experimental scheme. Syrian hamsters were vaccinated as indicated and challenged with SARS-CoV-2 (1 × 10^5^ p.f.u. SARS-CoV-2 Delta). Prime and prime-boost experiments were performed independently. **b**, Body weights (in %) after virus challenge were measured until analysis timepoint and displayed according to vaccination group. Violin plot (truncated) with quartiles and median. **c**–**l**, Results of prime-vaccinated and challenged animals (**c**–**e**,**i**,**j**) and results of prime-boost-vaccinated and challenged animals (**f**–**h**,**k**,**l**): number of genomic RNA (gRNA) copies detected in oropharyngeal swabs (**c**,**f**) and homogenized lung tissue (**d**,**g**). **e**,**h**, Quantification of replication-competent virus as p.f.u. per 50 mg homogenized lung tissue. Dotted line marks the limit of detection (DL = 5 p.f.u.). Titre below the detection limits set to DL/2 = 2.5 p.f.u. **i**,**k**, Lung inflammation was scored including severity of pneumonia, alveolar epithelial necrosis and endothelialitis. **j**,**l**, Lung oedema score accounting for perivascular and alveolar oedema. **m**, H&E-stained left lung sections illustrate different severities of pneumonia including peribronchial cuffs and consolidated areas between different vaccine schedules and non-vaccinated animals at 5 dpc. Scale bar, 3 mm. In **c**–**e** and **f**–**h** scatter dot plots: lines indicate means, symbols represent individual hamsters. In **i**,**j**,**k**,**l**: centre lines represent medians, boxes the 25th to 75th percentiles, and whiskers the minimum to maximum values; symbols represent individual hamsters. In **c**–**l**, two-way analysis of variance (ANOVA) and Tukey’s multiple comparisons test are shown. *n* = 5 animals per group. *P < 0.05, **P < 0.01, ***P < 0.001 and ****P < 0.0001. Fig. 1a was created with BioRender.com. Source data

**Extended Data Fig. 1:**
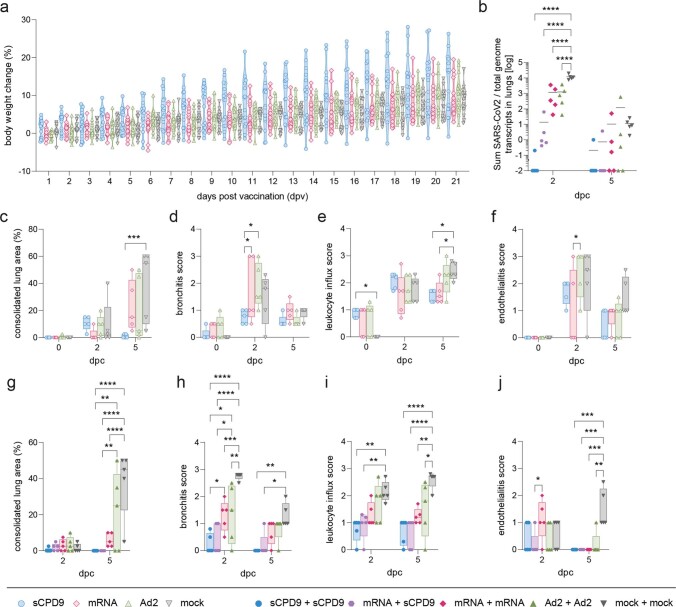
Body weight development post vaccination. (**a**) Body weight development in percent of prime vaccinated hamsters were measured for 21 days until virus challenge time point and displayed according to vaccination group. Violine plot (truncated) with quartiles and median. (**b**) Prime-boost experiment: Relative expression of SARS-CoV-2 total canonical junction-spanning viral mRNA transcripts, compared to the total genomic transcripts, generated after bulk RNA sequencing analysis from homogenized lung tissue. Values are shown in log10 scale for both time-points analyzed. Scatter dot plot with mean. Two-way ANOVA (analysis of variance) and Tukey’s multiple comparison test. (**c** – **j**) Semi-quantitative scoring of histopathological findings of Syrian hamsters included in prime (**c** – **f**) and prime-boost setting (**g** – **j**): (**c**, **g**) Consolidated lung area found in the left lung lobe is displayed in percentage. (**d**, **h**) Bronchitis score accounts for severity of bronchial epithelial necrosis and bronchitis. (**e**, **i**) To consider local cellular immune response, pulmonary infiltration of neutrophils, lymphocytes and macrophages was assessed in the leukocyte influx score. (**f**, **j**) The extent of endothelialitis in the left lung lobe is indicated in the endothelialitis score. (**c** – **j**) Results are shown as centre (median), box (25^th^ to 75^th^ percentile) and whiskers (Min to Max.) plots. Two-way ANOVA and Tukey’s multiple comparison test. ∗p < 0.05, ∗∗p < 0.01, ∗∗∗p < 0.001, and ∗∗∗∗p < 0.0001. (**b** – **j**) n = 5 individual hamsters for all groups. Prime and prime-boost experiment were conducted independently. Source data

In a heterologous SARS-CoV-2 Delta challenge setting, we evaluated efficacy and mode of action of the commercial mRNA vaccine BNT162b2 and two vaccine candidates, Ad2-Spike, an adenoviral vector carrying the spike glycoprotein of SARS-CoV-2^23^ and a live-attenuated SARS-CoV-2 named sCPD9^24,25^. To assess efficacy, Syrian hamsters were vaccinated with a single dose (prime-only regimen) and challenged with SARS-CoV-2 Delta variant 21 d post vaccination. Another group of hamsters received two vaccine doses 21 d apart (prime-boost regimen) and were challenge-infected 14 d after boosting (Fig. 1a). All vaccinations were well tolerated, as evidenced by steady weight gains post vaccination (Extended Data Fig. 1a).

### Vaccination alleviates clinical symptoms and reduces virus load

All vaccination strategies protected hamsters from SARS-CoV-2 infection-induced body weight loss (Fig. 1b). Following a single vaccination, none of the vaccines completely prevented infection by SARS-CoV-2 Delta as evidenced by the presence of viral RNA in respiratory tracts (Fig. 1c,d). Only sCPD9 vaccine effectively reduced replicating virus to undetectable levels 2 d post challenge (dpc) (Fig. 1e). Prime-boost vaccination improved overall vaccine efficacy against SARS-CoV-2 (Fig. 1f,g). Following prime-boost vaccination, viral RNA was significantly reduced, yet still detectable in all groups in oropharyngeal swabs and lungs. Vaccination schemes using sCPD9 were superior in reducing viral RNA (Fig. 1f,g). Similarly, levels of replication-competent virus in lungs were significantly reduced in vaccinated animals at 2 dpc. Importantly, only sCPD9 booster vaccination reduced replicating virus levels below the detection threshold, regardless of heterologous (mRNA) or homologous (sCPD9) priming (Fig. 1h). Results were confirmed by sequencing of bulk RNA from lungs (Extended Data Fig. 1b).

### LAV is superior in preventing inflammatory lung damage

**Fig. 2:**
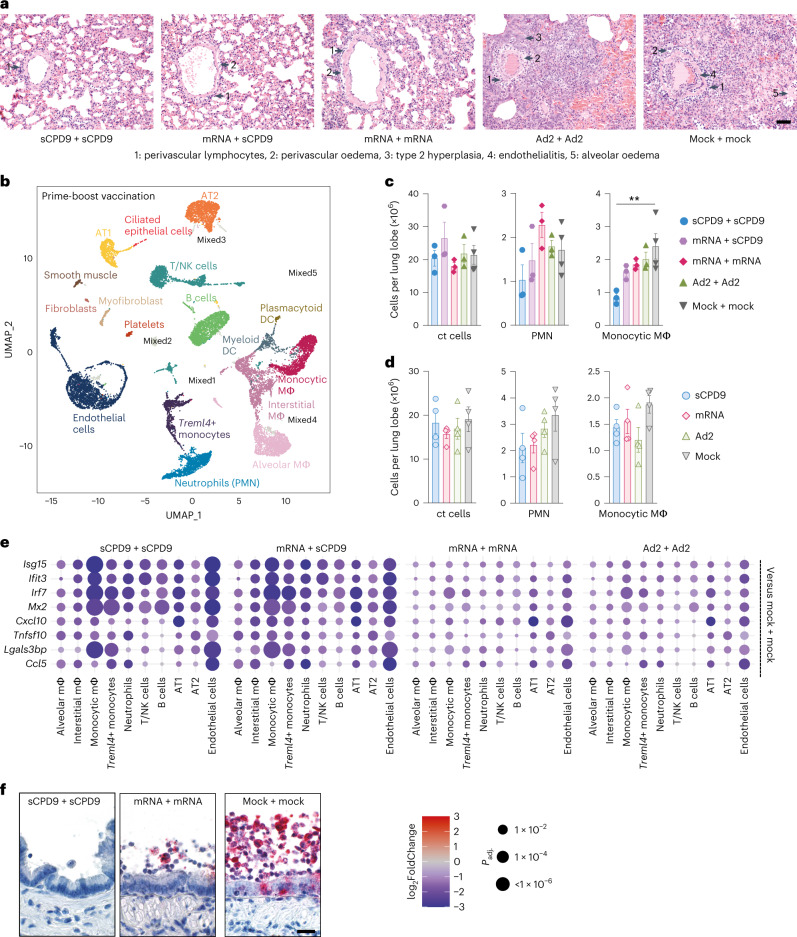
Pneumonia and pro-inflammatory transcriptional response are strongly reduced in vaccinated animals. **a**, H&E-stained lung sections at 5 dpc from the prime-boost vaccination experiment identified perivascular lymphocytes (1), perivascular oedema (2), metaplastic epithelial remodelling (3), endothelialitis (4) and alveolar oedema (5) as labeled by numbered arrows, groups as indicated. Scale bar, 90 µm. **b**, Two-dimensional projections of single-cell transcriptomes using uniform manifold approximation projection (UMAP) of lung cells (prime-boost experiment). Cells are coloured by cell type as annotated on the basis of known marker genes. **c**,**d**, Manual cell count of isolated lung cells per lung lobe (ct cells), calculated numbers of indicated cell types (PMN, monocytic macrophages) based on scRNA-seq-determined cell frequencies for the prime-boost experiment (**c**) and prime experiment (**d**). Bar plots with mean ± s.e.m., symbols represent individual hamsters (*n*_sCPD9_ = 4, *n*_mRNA_ = 4, *n*_Ad2_ = 4, *n*_mock_ = 4, *n*_sCPD9+sCPD9_ = 3, *n*_mRNA+sCPD9_ = 3, *n*_mRNA+mRNA_ = 3, *n*_Ad2+Ad2_ = 3, *n*_mock+mock_ = 4), ordinary one-way ANOVA and Tukey’s multiple comparisons test, **P < 0.01. **e**, Dot plots showing fold changes of gene expression in indicated cell types of the four prime-boost vaccination strategies compared to mock-mock-vaccinated animals. Selected interferon-stimulated genes and pro-inflammatory cytokines are visualized as follows: coloration and point size indicate log_2_-transformed fold changes (FC) and *P* values, respectively, in vaccinated compared to mock-mock-vaccinated animals. Adjusted *P* values (*P*_adj_) were calculated by DEseq2 using Benjamini–Hochberg corrections of two-sided Wald test *P* values. Genes are ordered by unsupervised clustering. **f**, Localization of viral RNA by in situ hybridization in a longitudinal section of a bronchus at 2 dpc. Red signals, viral RNA; blue, hemalum counterstain. Scale bar, 30 µm. Source data

**Extended Data Fig. 2:**
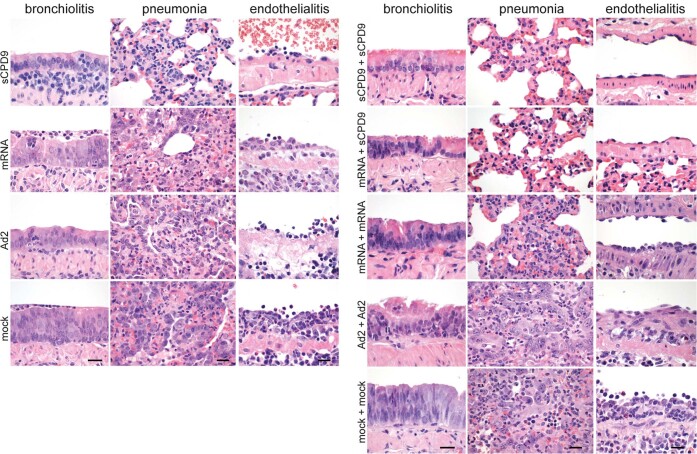
Lung histopathology. Representative histopathology of haematoxylin and eosin-stained lung sections from prime-only and prime-boost vaccination experiments at 5 dpc. Images are representative of n = 5 hamsters per indicated group. Prime and prime-boost experiments were performed independently. For both experiments, columns show, from left to right, bronchitis, pneumonia affecting the respiratory alveoli and blood vessels with endothelialitis. In the prime-only approach, and in contrast to all other groups that developed necrosupperative and hyperplastic bronchitis, only sCPD9 vaccinated hamsters had negligible bronchitis in the presence of BALT-like subepithelial infiltration with lymphocytes and plasma cells. In the lungs, alveoli of sCPD9 vaccinated animals presented with much less consolidated respiratory parenchyma, with less infiltrating macrophages and neutrophils. Only Ad2 and mock- vaccinated animals developed marked alveolar metaplastic remodeling, indicating regeneration after necrosis of alveolar epithelia. Endothelialitis was milder in all vaccinated groups compared to mock-vaccinated animals. In the prime-boost experiments, hyperplastic bronchitis was mildest in sCPD9+sCPD9 and mRNA+sCPD9 vaccinated hamsters. Consolidation of respiratory parenchyma and alveolitis were least severe in both sCPD9 boostered groups. Metaplastic epithelial remodeling was particularly pronounced in Ad2-Ad2 vaccinated animals. Endothelialitis was strongly reduced to similar degrees in all boostered groups. Scale bars = 20 μm for each column. Source data

To determine infection-induced lung damage, challenged hamsters were examined by histopathology. After single vaccination, sCPD9 was most efficient in preventing inflammation and pneumonia, as evidenced by lesser consolidated lung areas (Fig. 1m) and lower scores for lung inflammation, bronchitis and oedema (Fig. 1i,j and Extended Data Fig. 1c–f). Notably, animals that received other vaccination schedules displayed more prominent bronchial hyperplasia (Extended Data Fig. 2). A similar trend was observed for prime-boost regimens; however, particularly the mRNA vaccine displayed an improved histological outcome resulting from homologous boost (Fig. 1k,l and Extended Data Fig. 1g–j). Overall, homologous sCPD9 prime-boost vaccination provided superior lung protection from inflammation (Figs. 1m and 2a, and Extended Data Fig. 2). Lung transcriptome analysis also showed a broad downregulation of infection- and inflammation-related genes in vaccinated hamsters, with the greatest effects seen in homologous and heterologous sCDP9 vaccinations (Supplementary Fig. 1).

To correlate levels of inflammation with cellular responses, we performed single-cell RNA sequencing (scRNA-seq) of lung samples (Fig. 2b and Supplementary Fig. 2a). Results showed that pulmonary recruitment of monocytic macrophages was significantly reduced in sCPD9 + sCPD9-vaccinated animals at 2 dpc (Fig. 2c and Supplementary Fig. 2b). Similar, although less pronounced effects, were observed in animals that received sCPD9 prime-only vaccination (Fig. 2d and Supplementary Fig. 2c). Additionally, interferon-stimulated genes induced by SARS-CoV-2 infection^26^ were broadly downregulated in vaccinated compared with unvaccinated animals, with monocytic macrophages, *Treml4*^*+*^ monocytes and endothelial cells appearing particularly responsive (Supplementary Fig. 3). Inflammatory mediators such as *Cxcl10* or *Tnfsf10* showed a more uniform response pattern across cell types compared with interferon-stimulated genes (Fig. 2e and Supplementary Fig. 4a). Since macrophage subtypes showed different gene expression patterns between vaccination groups, we examined the distribution of viral RNA within lungs by in situ hybridization (Fig. 2f and Supplementary Fig. 4b). In unvaccinated animals, viral RNA was detected throughout the lungs, while mRNA+mRNA vaccination reduced its occurrence to single patches. In sCPD9 + sCPD9 animals, viral RNA was barely detectable (Supplementary Fig. 4b). Notably, in mRNA+mRNA animals, most of the detectable viral RNA was present in macrophages (Fig. 2f).

### LAV elicits the most potent humoral immunity against SARS-CoV-2

**Fig. 3:**
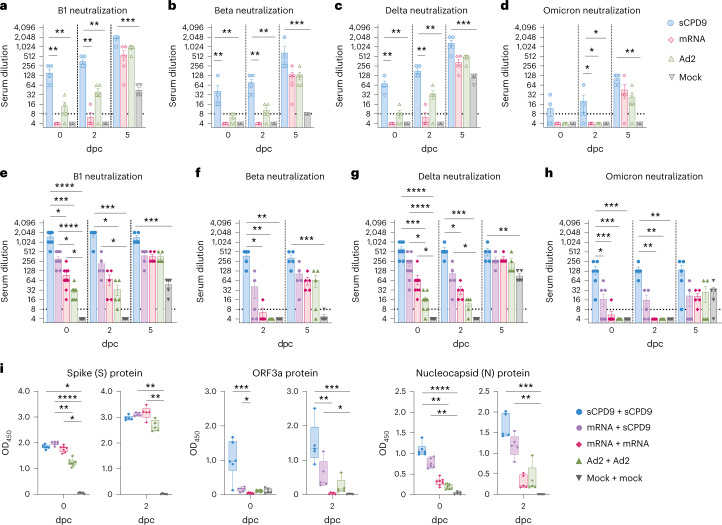
Antibody and blood cellular immune response to vaccination and challenge. **a**–**h**, Serum neutralization titres of hamsters that received prime (**a**–**d**) and prime-boost (**e**–**h**) vaccination before (0 dpc) and at 2 or 5 dpc with SARS-CoV-2. Neutralization capacity was tested against variant B1 (**a**,**e**), Beta (**b**,**f**), Delta (**c**,**g**) and Omicron BA.1 (**d**,**h**). The lower limit of detection was at dilution 1:8 (dotted line) and the upper limit at 1:2,048. Bar plots with mean ± s.e.m., *n* = 5 per condition; in **e**–**h**, *n* = 10 at baseline (0 dpc), except in **e** and **g** where *n*_sCPD9+sCPD9 (0dpc)_ = 9 and in **h** where *n*_sCPD9+sCPD9 (0dpc)_ = 6, *n*_mRNA+sCPD9 (0dpc)_ = 9, *n*_mRNA+mRNA (0dpc)_ = 8, *n*_Ad2+Ad2(0dpc)_ = 8 and *n*_mock+mock (0dpc)_ = 7 due to limited serum quantities. **i**, SARS-specific IgG levels against spike, ORF3a and nucleocapsid protein in sera from prime-boost-vaccinated hamsters on days 0 and 2 after challenge. Results are shown as optical density (OD) determined at 450 nm. Centre line, median; box, 25th to 75th percentiles; whiskers, minimum to maximum; symbols indicate individual values, *n*_sCPD9+sCPD9 (day 0)_ = 6, *n*_mRNA+sCPD9 (day 0)_ = 6, *n*_mRNA+mRNA (day 0)_ = 7, *n*_Ad2+Ad2 (day 0)_ = 8, *n*_mock+mock (day 0)_ = 5, *n*_sCPD9+sCPD9 (day 2)_ = 5, *n*_mRNA+sCPD9 (day 2)_ = 5, *n*_mRNA+mRNA (day 2)_ = 5, *n*_Ad2+Ad2 (day 2)_ = 5 and *n*_mock+mock (day 2)_ = 5 animals. For **a**–**i**, Kruskal-Wallis and Dunn’s multiple comparisons tests were performed; *P < 0.05, **P < 0.01, ***P < 0.001 and ****P < 0.0001. Source data

To determine humoral responses, we quantified the ability of hamster sera collected before challenge (0 dpc) and at 2 and 5 dpc of prime (Fig. 3a–d) and prime-boost (Fig. 3e–h) vaccinated hamsters to neutralize SARS-CoV-2 variants. The capacity of sera from sCPD9 vaccinees to neutralize ancestral SARS-CoV-2 variant B.1 significantly exceeded that of all other groups (Fig. 3a). Similarly, sCPD9 sera provided superior neutralization of variants of concern B.1.351 (Beta), B.1.617.2 (Delta) and B.1.1.529 (Omicron, BA.1) (Fig. 3b,c). For Omicron BA.1, neutralization capacity was considerably reduced in all groups; however, neutralization by sCPD9 sera was significant (Fig. 3d). Generally, challenge infection increased neutralizing antibodies over time in all groups by 5 dpc (Fig. 3a–d).

Hamsters boosted with sCPD9 or the mRNA vaccine produced measurably more neutralizing antibodies than those receiving prime-only vaccination. Overall, booster vaccination increased serum neutralization capacity across different variants (Fig. 3e–h). Among the tested variants, Omicron BA.1 displayed the greatest ability to escape neutralization. Only prime-boost vaccination with sCPD9 provided hamsters with a significant ability to neutralize Omicron BA.1 (Fig. 3h).

Prime-boost sCDP9 and mRNA+sCDP9-vaccinated hamsters mounted significant IgG antibody responses against spike, ORF3a and nucleocapsid protein (N), whereas IgG from prime-boost mRNA and Ad2-vaccinated hamsters only reacted with spike protein (Fig. 3i). Although antibodies directed against N and ORF3a are unlikely to contribute to virus neutralization, abundance of these antibodies illustrates the broader immunological response elicited by LAV vaccination. Higher virus neutralization titre in animals that underwent prime-boost vaccination (compare Fig. 3a–d with e–h) as well as increased IgG anti-spike reactivity following a challenge infection of these animals (Supplementary Fig. 4c) suggest a benefit of booster vaccinations.

### LAV favours adaptive cellular immune responses in blood

**Fig. 4:**
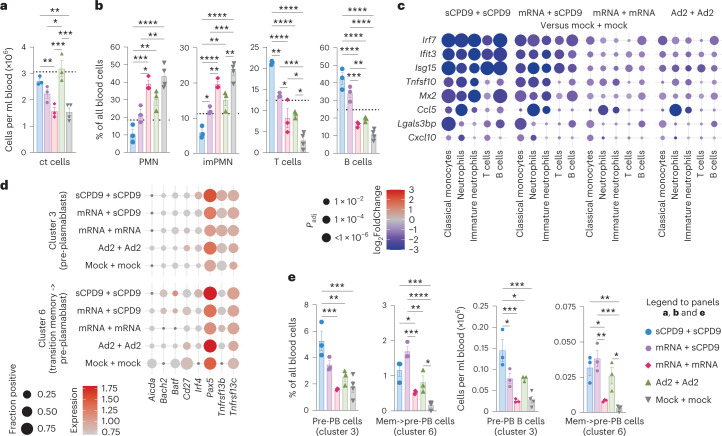
Cellular immune response to vaccination and challenge in blood. **a**–**e**, Analysis of cellular composition and gene expression by scRNA-seq at 2 dpc in blood of prime-boost-vaccinated hamsters. **a**, Manual count of cells per ml blood. **b**, Frequencies of indicated cell types among blood cells. Dotted lines mark the mean levels found in naïve hamsters (*n* = 3, naïve hamster data derived and reprocessed from ref. ^26^). Bar plots with mean ± s.e.m., *n* = 3. One-way ANOVA and Tukey’s multiple comparisons test were conducted. **c**, Dot plots showing fold changes of gene expression in indicated cell types of the four prime-boost vaccination strategies compared to mock-mock-vaccinated animals. Selected interferon-stimulated genes and pro-inflammatory cytokines are visualized as follows: coloration and point size indicate log_2_-transformed FC and *P* values, respectively, in vaccinated compared to mock-mock-vaccinated animals. *P*_adj_ were calculated by DEseq2 using Benjamini–Hochberg corrections of two-sided Wald test *P* values. Genes are ordered by unsupervised clustering. **d**, Dot plots showing expression of selected B-cell development marker genes in the blood B-cell subclusters shown in Supplementary Fig. 9a. The size of the dot represents the fraction of cells in which at least one unique molecular identifier (UMI) of the respective gene was detected, while the colour is proportional to the average expression in those cells. **e**, Frequencies and numbers of pre-plasmablast (pre-PB) identified in B-cell cluster 3 and memory to pre-plasmablast transitioning cells (mem->pre-PB) identified in B-cell cluster 6. Bar plots with mean ± s.e.m., *n* = 3. One-way ANOVA and Tukey’s multiple comparisons tests were performed; *P < 0.05, **P < 0.01, ***P < 0.001, ****P < 0.0001. Source data

**Extended Data Fig. 3:**
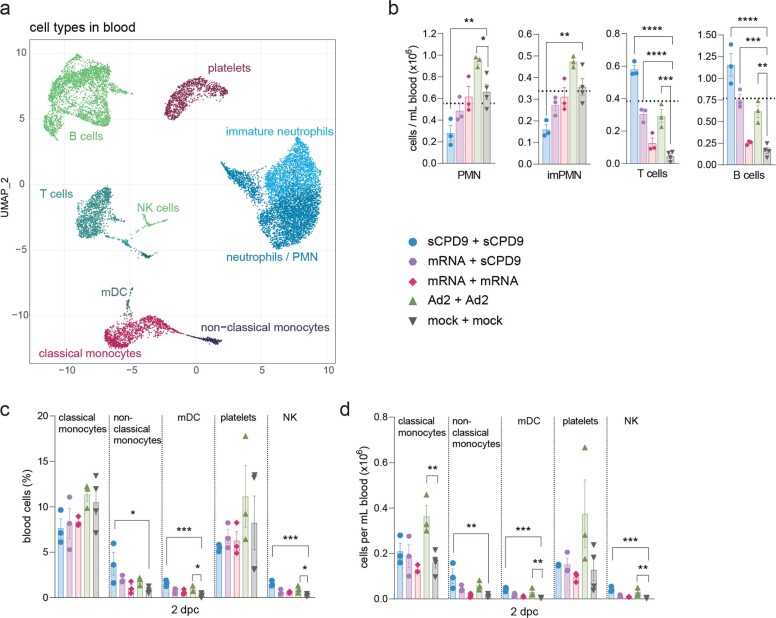
Immune cell responses in blood. (**a**) Two-dimensional projections of single-cell transcriptomes using UMAP of blood cells from prime-boost experiment. Cells are colored by cell types as annotated based on known marker genes. (**b**, **d**) Numbers of cellular components per ml blood for the prime-boost experiment. (**c**) Percentage of blood cellular components for the prime-boost experiment. (**b** – **d**) One-way ANOVA and Dunnett’s multiple comparisons test against mock-mock group per cell type are shown. Mean ± SEM, symbols represent individual hamster. n_sCPD9 + sCPD9_ = 3, n_mRNA + sCPD9_ = 3, n_mRNA + mRNA_ = 3, n_Ad2 + Ad2_ = 3, n_mock + mock_ = 4 individual hamsters as symbols. ∗p < 0.05, ∗∗p < 0.01, and ∗∗∗p < 0.001. Source data

Next, we evaluated blood single-cell immune responses to prime-boost vaccination (Extended Data Fig. 3a). Blood cell counting identified significantly higher cell densities in sCPD9- and Ad2-vaccinated prime-boost groups (Fig. 4a). Both relative and absolute cell numbers revealed substantial differences between vaccination strategies (Fig. 4b and Extended Data Fig. 3b–d). Frequencies of mature and immature neutrophils (imPMN), which are increased particularly in severe COVID-19^27^ and in SARS-CoV-2-infected Syrian hamsters^26^, were lowest for sCPD9 + sCPD9-vaccinated animals (Fig. 4b). In contrast, B, T and plasma cells followed the opposite trend and displayed highest abundancies following the sCPD9 + sCPD9 regime (Fig. 4b and Extended Data Fig. 3b). Concordant with our observations in lungs, genes related to infection and inflammation were broadly downregulated in myeloid cells of vaccinated animals (Fig. 4c and Supplementary Fig. 5).

To investigate activation of vaccine-induced immune memory, we first examined single-cell transcriptomes of circulating B cells, with a focus on B and plasma cells of prime-boost-vaccinated hamsters. Subclustering yielded 8 populations (Supplementary Fig. 6a), which were assigned to cellular states on the basis of known marker genes^28,29^. Of note, in the absence of surface marker information, these assignments probably remain incomplete. In our analysis, we therefore focused on differences in gene expression patterns and investigate whether a putative ‘memory recall gene expression signature’ would display differences. We first determined cluster 8 to probably represent plasmablasts/plasma cells due to the presence of, for example, *Prdm1* (which encodes Blimp-1) or *Irf4*. Cluster 3 featured intermediate *Prdm1* levels, as well as *Tnfrsf17* and *Tnfrsf13b*. Cluster 6 in comparison showed higher levels of *Pax5*, *Cd19*, *Cd27*, *Bach2* or *Aicda* and lower levels of *Prdm1*, *Xbp1* or *Spib* (Supplementary Fig. 6b). On the basis of these patterns, we assumed that these two clusters would contain (pre-) plasmablasts and would therefore be most interesting for investigating memory recall. Probing a set of genes involved in B-cell regulation, we found upregulation of *Irf4*, *Pax5* and *Tnfrsf13b/c* in cluster 3, while in cluster 6, *Bach2*, *Irf4*, *Pax5* and *Tnfrsf13b/c* were upregulated and *Aicda*, *Batf* and *Cd27* were downregulated (Fig. 4d and Supplementary Fig. 6c). This potential early ‘memory recall gene expression signature’ was strongest upon homologous or heterologous prime-boost vaccination with sCPD9, which induced the highest antibody titre (Fig. 3e–h). In line with this, clusters 3 and 6 cells were significantly more abundant in sCPD9 + sCPD9-vaccinated hamsters (Fig. 4e).

### LAV enhances T-cell proliferation in response to SARS-CoV-2 challenge

**Fig. 5:**
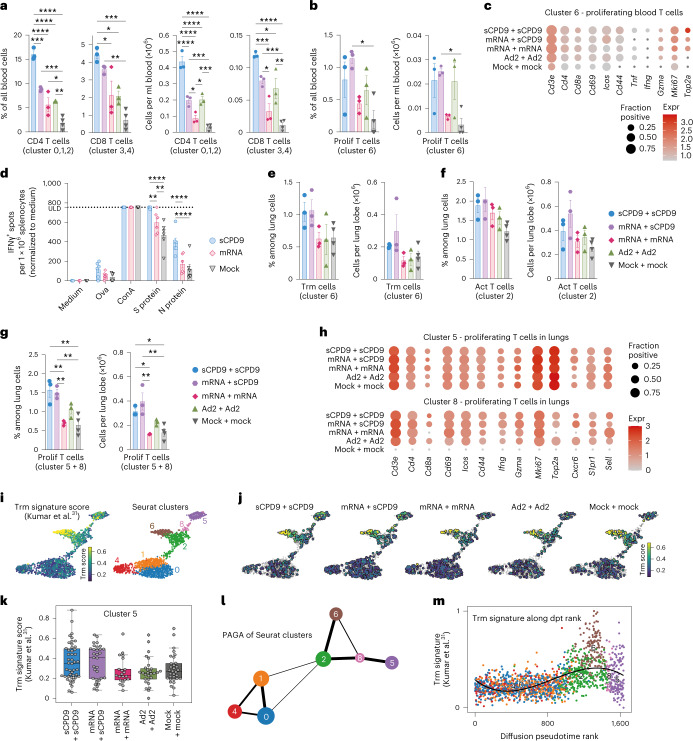
T-cell responses to vaccination and challenge. **a**–**k**, T-cell subsets by scRNA-seq (2 dpc) of prime-boost-vaccinated hamsters. Frequencies and numbers of (**a**) CD4 (cluster 0,1,2) and CD8 (cluster 3,4) T cells, and (**b**) proliferating T cells (cluster 6) in blood. Bar graph with mean ± s.e.m., *n* = 3 for all groups, except *n*_mock+mock_ = 4. Ordinary one-way ANOVA and Tukey’s multiple comparisons test. **c**, Dot plots showing expression of selected genes in blood cluster 6 (T and NK subcluster analysis in Supplementary Fig. 7a). Dot size represents fraction of cells with UMI > 1, colour indicates expression. **d**, IFN-γ ELISpot analysis 14 d post prime vaccination. Bar graph with mean ± s.e.m., *n* = 6, displaying spot counts normalized to medium stimulation for each animal, individually. Dotted line, upper limit of detection (ULD). Two-way ANOVA and Tukey’s multiple comparisons test. **e**–**g**, Frequencies and numbers of (**e**) tissue-resident memory T cells (Trm, cluster 6), (**f**) activated T cells (Act T, cluster 2) and (**g**) proliferating T cells (prolif T cells, cluster 5 + 8) in lungs. Ordinary one-way ANOVA and Tukey’s multiple comparisons test. Bar graph with mean ± s.e.m., *n* = 3 for all groups, except *n*_mock+mock_ = 4. In **a**,**b**,**d**–**g**: *P < 0.05, **P < 0.01, ***P < 0.001, ****P < 0.0001. **h**, Dot plots showing expression of selected genes in lungs, clusters 5 and 8 (T and NK subcluster analysis in Supplementary Fig. 9a). Dot size represents fraction of cells with UMI > 1, colour indicates expression. **i**,**j**, Trm gene set (refs. ^28,31^) signature score in cells from selected T-cell subclusters over all groups (**i**) and for individual groups (**j**), colour indicates signature score for Trm gene set. **k**, Trm signature score in cells of cluster 5. Centre, median; box, 25th to 75th percentiles; and whiskers, minimum to maximum values. Circles indicate individual analysed cells in cluster 5 pooled from *n* = 3 for all groups, except *n*_mock+mock_ = 4 animals. **l**, PAGA. Nodes represent clusters, edges represent extent of cluster connection, node size corresponds to cluster cell number and line thickness is proportional to connectivity. **m**, Trm signature (refs. ^28,31^) score as a function of diffusion pseudotime rank, with black line showing a polynomial fit of degree three. Source data

To investigate occurrence of T-cell memory recall, we proceeded with subclustering T and natural killer (NK) cells. To this end, we assayed CD4^+^, CD8^+^ and proliferating T cells in blood (Fig. 5a,b, and Supplementary Figs. 7 and 8). Analyses of gene expression indicative of proliferation (*Mki67*, *Top2a*), naïve or central memory status (*Sell*, *Ccr7*, *Lef1*, *Il7r*) and activation of T cells (*Cd69*, *Cd44*, *Klrg1*, *Icos*, *Cd40lg*) revealed that most blood T cells displayed either naïve or central memory phenotypes (cluster 0–4, Supplementary Fig. 7b–e). At 2 dpc, type 1-immunity effector genes (*Tbx21*, *Gzma*, *Gzmb*, *Faslg*, *Ifn*) were only expressed by blood NK cells (cluster 5, Supplementary Fig. 8). The proliferating T-cell population consisted of activated T cells expressing memory markers, such as *Il7r* (cluster 6, Supplementary Fig. 8). Proliferating T cells, albeit generally small in numbers, were significantly increased after heterologous vaccination (Fig. 5b). In line with this, the fraction of cells and expression level of proliferation-associated genes were highest when sCPD9 was included in the vaccination regimen (Fig. 5c). To determine T-cell antigen specificity, we vaccinated hamsters with either sCDP9 or mRNA and stimulated their splenocytes 14 d later with recombinant SARS-CoV-2 S or N protein. Interferon gamma (IFN-γ) enzyme-linked immunosorbent spot (ELISpot) served as readout. Spike protein stimulation induced IFN-γ-secreting cells for both vaccines, whereas upon stimulation with N protein, only splenocytes from sCPD9 vaccines secreted significantly more IFN-γ over mock, revealing superior and broader T-cell immunity upon LAV vaccination (Fig. 5d).

Next, we examined whether different prime-boost vaccination strategies differed in the ability to re-activate tissue-resident memory T cells (Trm) in lungs^30^. To characterize pulmonary T-cell subsets, we subclustered the initial T- and NK-cell clusters into 10 subclusters (Supplementary Fig. 9a). On the basis of *Nkg7*, *Cd3e*, *Cd4* and *Cd8a* gene expression, we assigned clusters 3, 7 and 9 as NK cells, cluster 4 as CD8^+^ T cells, clusters 0, 1, 2 and 6 as CD4^+^ T cells, and clusters 8 and 5 as proliferating T cells (*Mki67*, *Top2a*) (Supplementary Fig. 9b,c). Among CD4^+^ T-cell clusters, cells in cluster 2 displayed a mixed phenotype of effector, activation and memory gene markers (Supplementary Figs. 9b–e and 10a,b), and cells in clusters 0 and 1 were of naïve or central memory type (*Sell*, *Ccr7*, *Lef1*, *Il7r*, *Tcf7*, *S1pr1*) (Supplementary Fig. 10a). In cluster 6, we did not find genes associated with naïve or central memory-associated signatures (Supplementary Fig. 10a) but combined and strong expression of T-cell-homing and tissue retention genes (*Cxcr6*, *Rgs1*, *Prdm1*, *Znf683*, *Itga1* and *Itgae*), a signature indicative of Trm status (Supplementary Figs. 10b,c and 11). Trm cells (cluster 6) displayed higher gene expression level and cell fraction expressing *Cxcr6*, a prominent tissue homing receptor, in sCPD9-vaccinated groups, while lymph node retention receptor *S1pr1* was least detected (Supplementary Fig. 12a). Across activated T cells (cluster 2), gene expression of activation and effector genes was independent of previous vaccination (Supplementary Fig. 12a). At 2 dpc, Trm cells, activated and proliferating T-cell populations were small and represented less than 2% of all lung cells. Trm cells and activated T cells trended towards higher frequencies and numbers in challenged sCPD9 vaccinees, yet only proliferating T cells displayed significantly higher values (Fig. 5e–g). Notably, contrary to proliferating T cells in blood, their lung counterparts expressed higher levels of *Ifng* and *Gzma* (Fig. 5h).

Next, we scored the Seurat clusters for a published human Trm gene set^31^ and observed a subset of cells in cluster 5 (proliferating T cells) with a high Trm signature score (Fig. 5i). At 2 dpc, the Trm signature score in proliferating T cells (cluster 5) was remarkably higher in sCPD9 vaccinees (Fig. 5j,k). In cluster 8 (proliferating T cells), overall cell numbers were too low to generate interpretable scores, and no cells were identified from unvaccinated animals (Supplementary Fig. 12b). Using a partition-based graph abstraction (PAGA) approach^32^, we identified particularly strong connectivity between clusters 2, 8 and 5, and clusters 2 and 6, as well as a possible connection between clusters 6 and 8 (Fig. 5l). Ordering cells according to global expression similarity by diffusion pseudotime^33^ and plotting this rank against the Trm signature further corroborates a path between clusters 2 and 6, and clusters 8 and 5, which is accompanied by variable Trm-like gene expression (Fig. 5m and Supplementary Fig. 12c,d). Overall, these findings suggest that a subset of proliferating T cells is Trm recall-derived and activated in response to SARS-CoV-2 challenge infection in sCPD9-boosted hamsters.

### LAV induces superior mucosal immunity

**Fig. 6:**
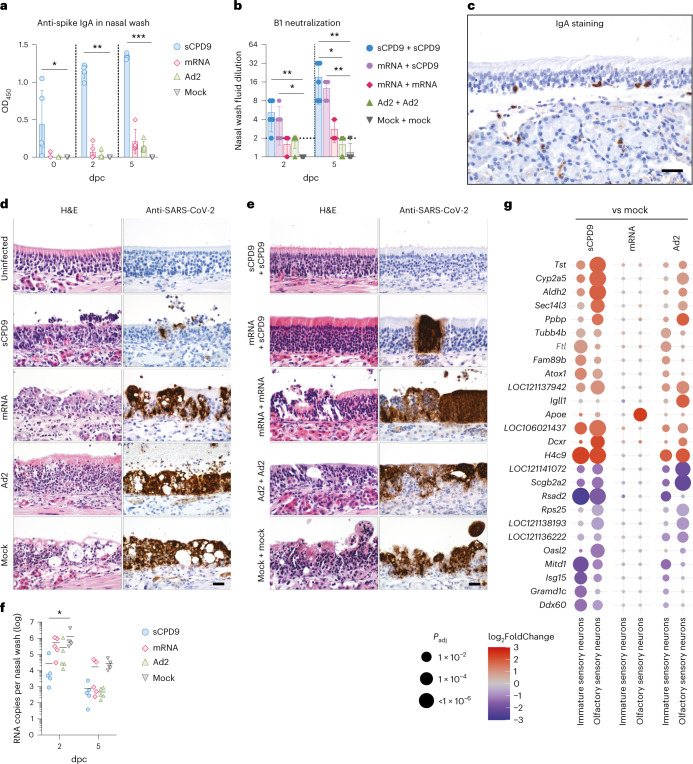
Protective effects on the mucosa and development of local immunity after vaccination. **a**, ELISA detecting anti-spike IgA levels in nasal washes of prime-vaccinated hamsters at indicated timepoints post challenge (dpc). Results display OD_450_. **b**, Neutralizing capacity against B.1 of nasal wash fluids from prime-boost-vaccinated hamster at indicated timepoints. Bar plots show mean ± s.d. Kruskal-Wallis and Dunn’s multiple comparisons test per timepoint; *P < 0.05, **P < 0.01, ***P < 0.001, ****P < 0.0001. Symbols indicate individual hamsters, *n* = 5 animals per group. **c**, Immunohistochemical staining of IgA in the olfactory epithelium and submucosal glands at 2 dpc. Scale bar, 60 µm. **d**, Longitudinal histopathological sections of olfactory epithelium, with H&E staining (left) and SARS-CoV-2 N protein immunohistochemistry (right) of the prime-only experiment at 2 dpc, with an additional section of an uninfected tissue. **e**, As in **d** but for the prime-boost vaccination experiment. Scale bar, 20 µm. In **c**–**e**, images are representative of *n* = 5 hamsters per indicated group. Prime and prime-boost experiments were performed independently. **f**, Virus RNA loads detected in nasal washes of prime-vaccinated hamsters at 2 and 5 dpc. In scatter dot plots, lines indicate means, symbols represent individual hamsters. *n* = 5. Two-way ANOVA and Tukey’s multiple comparisons test; *P < 0.05. **g**, Dot plots showing fold changes of gene expression in indicated cell types of the three prime vaccination strategies compared to mock-vaccinated animals. Selected interferon-stimulated genes and pro-inflammatory cytokines are visualized as follows: coloration and point size indicate log_2_-transformed FC and *P* values, respectively, in vaccinated compared to mock-vaccinated animals. *P*_adj_ were calculated by DEseq2 using Benjamini–Hochberg corrections of two-sided Wald test *P* values. Genes are ordered by unsupervised clustering. Source data

**Extended Data Fig. 4:**
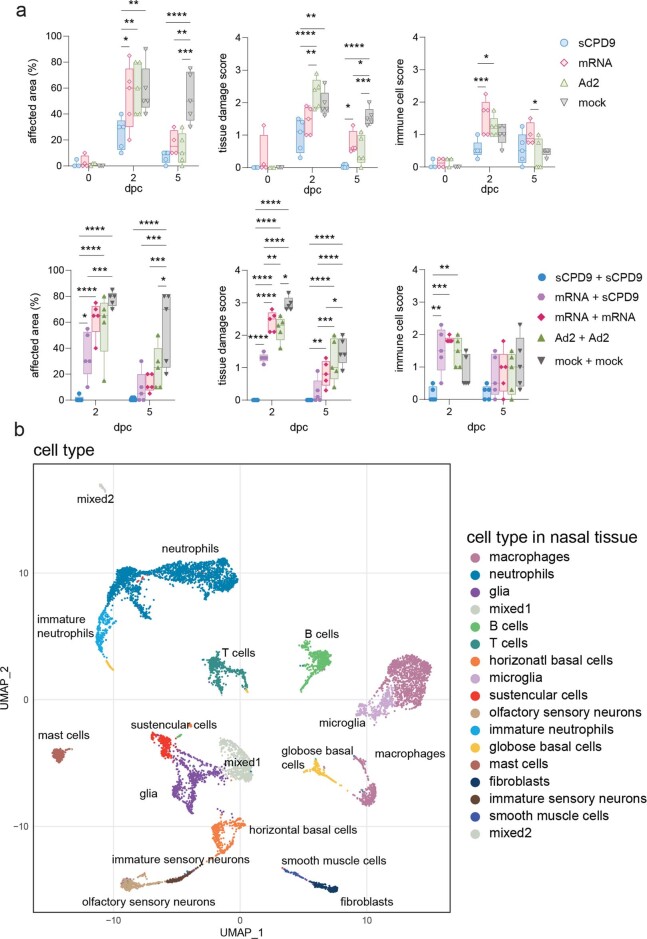
Mucosal immune responses. (**a**) Semi-quantitative scorings of histopathological findings in hamsters that received prime vaccination (top row) or prime-boost vaccination (bottom row). The area affected by mucosal damage due to SARS-CoV-2 infection is shown in percentage. The tissue damage score accounts for epithelial exfoliation, necrosis, apoptosis, cellular debris and cilium loss. Presence of neutrophils and lymphocyte in the nasal conchae is assessed by the immune cell score. Centre (median), Box (25th to 75th percentile) and whiskers (Min to Max). Two-way ANOVA and Tukey’s multiple comparison test were performed for statistical evaluation. N = 5 individual hamster per group. ∗p < 0.05, ∗∗p < 0.01, ∗∗∗p < 0.001, ∗∗∗∗p < 0.0001. (**b**) Two-dimensional projections of single-cell transcriptomes using UMAP of nasal tissue cells from prime experiment. Coloration indicates cell types as annotated based on known marker genes. Source data

**Extended Data Fig. 5:**
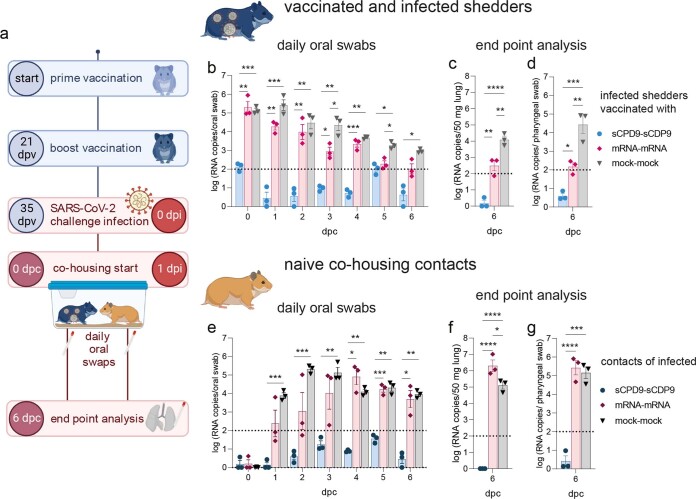
Onward transmission analysis. (**a**) Design of a proof-of-principle onward transmission experiment. Hamsters were vaccinated with a furin cleavage site deleted sCPD9 LAV or the BNT162b2 mRNA vaccine in a prime-boost schedule. Challenge infection was performed with SARS-CoV-2 variant B.1, vaccinated and challenged animals were co-habitated with naïve contacts starting 24 hours post challenge for 6 consecutive days. (**b**) SARS-CoV-2 RNA load in daily oral swabs taken from vaccinated shedders throughout the period of co-housing. N = 3 per group, mean ± SEM. Two-way ANOVA, Tukey’s multiple comparisons test. (**c**) SARS-CoV-2 RNA load in lungs and (**d**) pharyngeal swabs at termination of vaccinated shedder animals. N = 3, mean ± SEM, One-way ANOVA, Tukey’s multiple comparisons test. (**e**) SARS-CoV-2 RNA load in daily oral swabs taken from naïve contact animals throughout the period of co-housing. N = 3, mean ± SEM. Two-way ANOVA, Tukey’s multiple comparisons test. **(f**) SARS-CoV-2 RNA load in lungs and (**G**) pharyngeal swabs at termination of naïve contact animals. N = 3, mean ± SEM, One-way ANOVA, Tukey’s multiple comparisons test. ∗p < 0.05, ∗∗p < 0.01, ∗∗∗p < 0.001, ∗∗∗∗p < 0.0001. Panel **a** was created with BioRender.com. Source data

In addition to potent T-cell memory and humoral immunity, induction of protective mucosal immunity is a distinguishing property of LAVs administered at sites of virus entry^34^. To correlate induction of mucosal immunity with vaccine regimens, we measured SARS-CoV-2 spike-specific IgA levels and neutralization capacity of nasal washes. We found that prime-only sCPD9-vaccinated animals harboured considerably larger quantities of IgA in nasal washes before and after challenge (Fig. 6a). Challenge infection further boosted levels of SARS-CoV-2 spike-specific IgA antibodies in sCPD9-vaccinated animals and induced detectable quantities in mRNA- and Ad2-vaccinated animals. Microneutralization assays against SARS-CoV-2 (variant B.1) with nasal washes obtained from prime-boosted animals confirmed IgA measurements. sCPD9 vaccinees exhibited markedly higher neutralization capacities at 2 and 5 dpc (Fig. 6b). Accordingly, we identified IgA-positive lymphocytes in the nasal mucosa of vaccinated animals (Fig. 6c). Histopathological scoring indicated that sCPD9-vaccinated animals displayed fewer affected tissue areas, less damage and reduced immune cells recruitment (Fig. 6d,e and Extended Data Fig. 4a). sCDP9 vaccination significantly reduced viral RNA in nasal washes compared with mock at 2 dpc (Fig. 6f), correlating with reduced signal in immunohistochemistry staining for viral N protein (Fig. 6d). To further evaluate putatively beneficial effects of mucosal immunity, we resorted to scRNA-seq of nasal tissue cells. First, we annotated cell types on the basis of previously published marker genes (Extended Data Fig. 4b^35^). Particularly in neuronal cells, differential gene expression analysis showed that interferon-stimulated genes (*Isg15*, *Oasl2* or *Rsad2*) were less expressed in sCPD9 vaccinees (Fig. 6g and Supplementary Fig. 13). Notably, bystander responses of neuronal cells in the olfactory epithelium are connected to loss of smell after SARS-CoV-2 infection^36^. Moreover, we find evidence that LAV vaccination prevents SARS-CoV-2 transmission. Following challenge infection, LAV-vaccinated animals shed significantly lower virus quantities compared with mRNA-vaccinated individuals. In this setup, LAV vaccination was able to prevent SARS-CoV-2 transmission while mRNA vaccination was not (Extended Data Fig. 5). Taken together, we provide evidence that the sCPD9 LAV provides superior protection against SARS-CoV-2 in both the lower and upper airways, making it a promising candidate for further investigation in clinical trials.

## Discussion

Current COVID-19 vaccines are highly effective in preventing severe disease; however, infection with newly emerging variants is not prevented and virus loads can be high in vaccinated individuals^37^. To control virus transmission and limit symptomatic infection, mucosal immunity at the site of virus entry is thought to be of paramount importance^38–40^.

We here present a cross-platform vaccine comparison that includes a LAV, which we find elicits superior protection from SARS-CoV-2 infection especially at mucosal sites of virus entry. This agrees with previous preclinical COVID-19 vaccine studies using intranasal administration of LAV, protein-based or virus-vectored spike vaccines^24^ and efficient induction of mucosal immunity^41–44^. Our observations on improved immunity induced by heterologous prime-boost vaccination are in line with recent studies that combine systemic priming followed by an intranasal boost with adenovirus vector or mRNA vaccines^19,45^. Importantly, virus-neutralizing anti-SARS-CoV-2 IgA at the nasal mucosa of vaccinated animals are much higher in sCPD9-vaccinated animals. It is well known that mucosal IgA exerts various functions, such as blocking virus entry, preventing intracellular fusion of virus and endosomal membranes as well as inhibiting release of viruses from host cells^46^. Overall protection from virus replication, tissue damage and lung inflammation were significantly better in sCPD9-vaccinated animals. At the same time, antigen recognition was considerably broader in animals that had received sCPD9, and these benefits are probably a result of important hallmark features of LAV. These include administration via the natural route of infection, presentation of the full antigenic repertoire of the virus and replication mimicking the target pathogen. Moreover, active replication of LAVs may cause prolonged and increased presentation of viral antigens compared with non-replicating vaccines—a factor that could contribute to the better efficacy observed here. In a small-scale experiment, LAV vaccination was able to abrogate onward transmission of SARS-CoV-2, while mRNA vaccination had only minor effects on transmission. The scRNA-seq analysis of samples from blood, lungs and nasal mucosa of vaccinated and SARS-CoV-2 challenge-infected hamsters revealed that across all important parameters, effects were strongest for sCPD9 vaccination in a prime-only setting. Similarly, in a prime-boost setting, double sCPD9 vaccination was superior to mRNA-sCPD9 vaccination, followed by double mRNA vaccination and double adenovirus vaccination. sCPD9-vaccinated animals had substantially reduced induction of pro-inflammatory gene expression programmes—a main feature of COVID-19 pathogenesis^47^. This was specifically true for cells of the innate immune system, such as monocytes and macrophages, which typically have strong pro-inflammatory transcriptional responses upon SARS-CoV-2 uptake^26^. If translatable to humans, this could mean a much higher chance for a mild or asymptomatic course of disease even in the case of infection with heterologous SARS-CoV-2 variants.

Additionally, we detected several gene expression signatures related to activation of adaptive immune memory. The enhanced development towards pre-plasmablasts derived from memory B cells and enhanced T-cell proliferation in the blood of challenged animals by scRNA-seq point towards rapid activation of memory cells^48^. We also detected significantly increased numbers of proliferating T cells in lungs of hamsters that received sCPD9. A subset of these proliferating T cells shared a Trm-specific signature and showed connectivity to the identified Trm cluster. One possible explanation for this observation is that SARS-CoV-2-specific tissue-resident memory T-cell seeding is enhanced following sCPD9 vaccination, which may enable faster local recall responses, characterized by enhanced proliferating T cells in corresponding vaccine groups. LAVs mimic natural infection, which is known to induce SARS-CoV-2-specific CD4^+^ Th1 cells secreting IFN-γ^49^. We detected IFN-γ upregulation in proliferating pulmonary T cells, indicating that SARS-CoV-2 challenge triggered a Th1 effector cell type response, and IFN-γ ELISpot analysis revealed multi-antigen reactivity in splenocytes of LAV-vaccinated hamsters. While mucosal IgA induction remains most important in limiting infection and thus transmission, airway memory CD4^+^ T cells contribute to protection against other coronaviruses^30^ and potentially enhance the antigenic repertoire recognized in a mucosal SARS-CoV-2 vaccine. Similarly, earlier studies using ovalbumin antigens and a combination of different vaccination routes indicated that not just IgA but also general Th1-mediated immunity is enhanced upon mucosal delivery^50^.

Our single-cell RNA-sequencing analysis has several limitations. This technique, as employed here, cannot fully capture processes such as reactivation of memory cells due to lack of surface markers and cell type-specific enrichment. Due to incomplete annotation of the Syrian hamster genome, we were not able to identify IgA-positive cells. Data quality of nasal mucosa cells was comparatively low due to the difficult dissociation of the tissue, which limited our observations at the site of initial infection. The data we present on the effect of vaccination on challenge virus transmission are preliminary and require larger-scale studies, use of more recent SARS-CoV-2 variants and mechanistic analyses for validation. While our data show superiority and therefore promise for further development and refinement of LAVs, there is a caveat for extrapolating the results of preclinical animal trials to the situation in humans. Clearly, clinical studies regarding safety and efficacy of live-attenuated vaccines are mandated to realistically assess the potential of these vaccines to combat the yet ongoing pandemic.

One issue with LAVs is their potential susceptibility to previously established immunity^51^, which would restrict vaccine virus replication and potentially limit their use as booster after initial immunization by vaccination or natural infection. We show here that sCPD9 does effectively boost immune responses and greatly improves protection when applied three weeks after initial vaccination. Importantly, sCPD9 enhances humoral immune responses, especially against known immune escape variants such as Beta and Omicron BA.1, while also improving the virological outcome of a heterologous challenge infection when applied as a booster three weeks after initial vaccination. This indicates a wide scope for the use of LAVs in populations that exhibit a high degree of baseline immunity induced by previous vaccination or infection.

Due to its high safety profile, sCPD9 was recently downgraded from biosafety level (BSL) 3 to BSL 2 by the relevant German state authority^52^. This is a key step towards clinical application of a SARS-CoV-2 LAV as it will facilitate production of a clinical grade vaccine and greatly ease clinical trials in humans.

## Methods

### Ethics statement

In vitro and animal work were conducted under appropriate biosafety conditions in a BSL-3 facility at the Institut für Virologie, Freie Universität Berlin, Germany. All animal experiments were performed in compliance with relevant institutional, national and international guidelines for the care and humane use of animals and approved by the competent state authority, Landesamt für Gesundheit und Soziales, Berlin, Germany (permit number 0086/20).

### Cells

Vero E6 (obtained from ATCC, CRL-1586), Vero E6-TMPRSS2 (obtained from the National Institute for Biological Standards and Control (NIBSC), 100978) and Calu-3 (obtained from ATCC, HTB-55) cells were cultured in minimal essential medium (MEM) containing 10% fetal bovine serum, 100 IU ml^−1^ penicillin G and 100 µg ml^−1^ streptomycin at 37 °C and 5% CO_2_. In addition, the cell culture medium for Vero E6-TMPRSS2 cells contained 1,000 µg ml^−1^ geneticin (G418) to ensure selection for cells expressing the genes for neomycin resistance and TMPRSS2.

### Viruses

The modified live-attenuated SARS-CoV-2 mutant sCPD9 and SARS-CoV-2 variants B.1 (BetaCoV/Munich/ChVir984/2020; B.1, EPI_ISL_406862), Beta (B.1.351; hCoV-19/Netherlands/NoordHolland_20159/2021) and Delta (B.1.617.2; SARS-CoV-2, Human, 2021, Germany ex India, 20A/452R (B.1.617)) were propagated on Vero E6-TMPRSS2 cells. Omicron BA.1 (B.1.1.529.1; hCoV-19/Germany/BE-ChVir26335/2021, EPI_ISL_7019047) was propagated on CaLu-3 cells. All virus stocks were whole genome sequenced before infection experiments to confirm genetic integrity in the majority of the population, specifically at the furin cleavage site. Before experimental infection, virus stocks were stored at −80 °C.

### Animal husbandry

Nine- to 11-week-old Syrian hamsters (*Mesocricetus auratus*; breed RjHan:AURA) were purchased from Janvier Labs and were housed in groups of 2 to 3 animals in individually ventilated cages. The hamsters had free access to food and water. They were allowed to get used to the housing conditions for 7 d before vaccination. For both experiments, the cage temperatures were maintained at a constant range of 22 to 24 °C with a relative humidity between 40 and 55%.

### Vaccination and infection experiments

For infection experiments, Syrian hamsters were randomly assigned to groups, with 50–60% of the animals in each group being female. In the first experiment, 15 hamsters were mock-vaccinated or vaccinated with live-attenuated sCPD9 virus, Ad2-spike or mRNA. Vaccination with sCPD9 was applied by intranasal instillation under anaesthesia (1 × 10^5^ focus-forming units (f.f.u.), 60 µl)^53^. Ad2-spike (5 × 10^8^ infectious units, 200 μl) and mRNA vaccine (5 μg mRNA, 100 μl) were applied intramuscularly. Mock-vaccinated hamsters were vaccinated by intranasal instillation with sterile cell culture supernatant obtained from uninfected Vero E6-TMPRSS2 cells. At 21 d after vaccination, hamsters were challenge-infected with SARS-CoV-2 Delta variant (1 × 10^5^ plaque-forming units (p.f.u.), 60 µl) by intranasal instillation under anaesthesia. In the second experiment, 10 hamsters were either mock-vaccinated or vaccinated with one of the three vaccines (see above) followed by a booster vaccination 21 d later. At 14 d after booster vaccination, the hamsters were challenged as described above.

### Transmission experiments

To determine onward transmission of challenge virus in vaccinated individuals, we vaccinated 3 animals per group in a prime-boost regimen. To this end, hamsters received either 1 × 10^4^ f.f.u. sCPD9delFCS in 60 µl MEM intranasally, 5 μg BNT162b2 mRNA in 100 μl normal saline (0.9% NaCl in sterile water) intramuscularly or 60 µl plain MEM intranasally (mock). Vaccination was boosted using the same vaccines for each respective group 21 d after initial vaccination.

Vaccinated hamsters were challenge-infected with 1 × 10^5^ p.f.u. SARS-CoV-2 variant B.1 as described above. At 24 h after infection, infected vaccinated hamsters were brought into contact with naïve animals and co-habitated to monitor transmission for 6 consecutive days. Daily oral swabs were obtained from each animal to monitor virus shedding and transmission.

### Vaccine preparations

sCPD9 was grown on Vero E6-TMRSS2 cells and titrated on Vero E6 cells as described previously; final titres were adjusted to 2 × 10^6^ f.f.u. ml^−1^ in MEM. Recombinant Ad2-spike was generated, produced on 293T cells and purified as previously described^23^. BNT162b2 was obtained as a commercial product (Comirnaty) and handled exactly as recommended by the manufacturer, except that the final concentration of mRNA was adjusted to 50 µg ml^−1^ (100 µg ml^−1^ is the recommended concentration for use in humans) by adding injection-grade saline (0.9% NaCl in sterile water) immediately before use.

To increase genetic stability of the sCPD9 construct, the furin cleavage site (FCS) of the spike protein was deleted to create sCPD9delFCS. This FCS-deleted vaccine virus was only used for the transmission study of this paper (Extended Data Fig. 5). Importantly, all vaccines used in this study contain the same SARS-CoV-2 spike antigen derived from the ancestral B.1 (Wuhan) sequence.

### Vaccination

sCPD9 was administered intranasally under general anaesthesia (0.15 mg kg^−1^ medetomidine, 2.0 mg kg^−1^ midazolam and 2.5 mg kg^−1^ butorphanol) at a dose of 1 × 10^5^ f.f.u. per animal in a total volume of 60 µl MEM. For transmission experiments (Extended Data Fig. 5), 1 × 10^4^ f.f.u. sCPD9delFCS was applied in the same way. Ad2-spike was injected intramuscularly at 5 × 10^8^ infectious units in 200 µl injection buffer (3 mM KCl, 1 mM MgCl_2_, 10% glycerol in PBS). BNT162b2 was injected intramuscularly at a dose of 5 µg mRNA per animal in 100 µl physiological saline (0.9% NaCl in sterile water).

### Nasal washes

Nasal washes were obtained from each hamster in this study. To this end, the skull of each animal was split slightly paramedian, such that the nasal septum remained intact on one side of the nose. Subsequently, a 200 µl pipette tip was carefully slid underneath the nasal septum and 150 µl wash fluid (PBS with 30 µg ml^−1^ ofloxacin and 10 µg ml^−1^ voriconazole) was applied. The wash was collected through the nostril and the washing procedure was repeated twice; approximately 100 µl of sample was recovered after the third wash.

Nasal washes obtained from the prime-only vaccination trial were subjected to enzyme-linked immunosorbent assay (ELISA) analysis of SARS-CoV-2 spike-specific IgA antibodies. Nasal washes obtained from the prime-boost vaccination trial were used for microneutralization assay to assess their capacity to neutralize the SARS-CoV-2 ancestral variant B1.

### Plaque assay

For quantification of replication-competent virus, 50 mg of lung tissue were used. Serial 10-fold dilutions were prepared after homogenizing the organ samples in a bead mill (Analytic Jena). The dilutions were plated on Vero E6 cells grown in 24-well plates and incubated for 2.5 h at 37 °C. Subsequently, cells were overlaid with MEM containing 1.5% carboxymethylcellulose sodium (Sigma Aldrich) and fixed with 4% formaldehyde solution 72 h after infection. To count the plaque-forming units, plates were stained with 0.75% methylene blue.

### Histopathology, immunohistochemistry and in situ hybridization

Lungs were processed as previously described^53^. After careful removal of the left lung lobe, tissue was fixed in PBS-buffered 4% formaldehyde solution (pH 7.0) for 48 h. For conchae preparation, parts of the left skull half were fixed accordingly. Afterwards, lungs or conchae were gently removed from the nasal cavity and embedded in paraffin, cut at 2 µm thickness and stained with hematoxylin and eosin (H&E). In situ hybridization on lungs was performed as previously described^54^ using the ViewRNA ISH Tissue Assay kit (Invitrogen by Thermo Fisher) according to the manufacturer’s instructions, with minor adjustments. For SARS-CoV-2 RNA localization, probes detecting N gene sequences (NCBI database NC_045512.2, nucleotides 28,274–29,533, assay ID: VPNKRHM) were used. Sequence-specific binding was controlled by using a probe for detection of pneumolysin. Immunohistochemistry on conchae was performed as described earlier^55^ (details in Supplementary Methods).

Blinded microscopic analysis was performed by a board-certified veterinary pathologist (J.B.).

### SARS-specific Ig measurement by ELISA from serum and nasal washes

An in-house ELISA was performed to investigate SARS-specific IgG levels in serum and SARS-specific IgA levels in nasal washes after vaccination (details in Supplementary Methods).

### Neutralization assays from nasal washes

To assess the capacity of nasal washes obtained from the prime-boost vaccination trial to neutralize authentic SARS-CoV-2 (B.1), nasal washes were diluted 1:1 in 2× MEM containing 50 µg ml^−1^ enrofloxacin and 10 µg ml^−1^ voriconazole. Subsequent serial dilutions were performed in MEM containing 25 mg ml^−1^ enrofloxacin, 5 µg ml^−1^ voriconazole and 1% FBS. SARS-CoV-2 (50 p.f.u.) were added to the nasal wash dilutions and dilutions from 1:2 to 1:256 were plated on near-confluent Vero E6 cells seeded in 96-well cell culture plates. At 3 d after inoculation, cells were fixed and stained with methylene blue. To identify virus-neutralizing dilutions, the integrity of the cell monolayer was assessed by comparison with control wells that contained either no nasal wash or no virus. The last dilution with no evidence of virus-induced cytopathic effect was considered the neutralizing titre for the respective sample.

### Serum neutralization assay

Serum samples were tested for their ability to neutralize different SARS-CoV-2 variants. Day 0 samples of the prime-boost trial could not be tested for neutralizing antibodies against B.1.351 (Beta) due to lack of material. Sera were inactivated at 56 °C for 30 min. Twofold serial dilutions (1:8 to 1:1,024) were plated on 96-well plates and 200 p.f.u. SARS-CoV-2 were pipetted into each well. After an incubation time of 1 h at 37 °C, the dilutions were transferred to 96-well plates containing sub-confluent Vero E6 cells and incubated for 72 h at 37 °C (B.1, Beta, Delta) or for 96 h at 37 °C (Omicron). The plates were fixed with 4% formaldehyde solution and stained with 0.75% methylene blue. Wells that showed no cytopathic effect were considered neutralized.

### IFN-γ ELISpot analysis

Hamster IFN-γ ELISpot analysis was performed as described previously^56^. In brief, the hamster IFN-γ ELISpot^BASIC^ kit (MABTECH) was used to detect IFN-γ secretion by 5 × 10^5^ isolated splenocytes, each in co-culture with different stimuli. Medium-treated splenocytes served as negative control and recombinant ovalbumin (10 mg ml^−1^) was used as negative protein control stimulus. General stimulation of T cells was achieved using 5 μg ml^−1^ concanavalin A (ConA, Sigma Aldrich). Recombinant SARS-CoV-2 (2019-nCoV) spike protein (S1 + S2 ECD, His tag; 10 mg ml^−1^; Sino Biological Europe) or 10 mg ml^−1^ recombinant SARS-CoV-2 (2019-nCoV) nucleocapsid protein (N) (Sino Biological Europe) were used to re-stimulate SARS-CoV-2-specific T cells. Spots were counted using an Eli.Scan ELISpot scanner (AE.L.VIS) and the analysis software ELI.Analyse v5.0 (AE.L.VIS).

### RNA extraction and qPCR

To quantify genomic copies in oropharyngeal swabs and 25 mg homogenized lung tissue, RNA was extracted using innuPREP Virus DNA/RNA kit (Analytic Jena) according to the manufacturer’s instructions. qPCR was performed using the NEB Luna Universal Probe One-Step RT–qPCR kit (New England Biolabs) with cycling conditions of 10 min at 55 °C for reverse transcription, 3 min at 94 °C for activation of the enzyme, and 40 cycles of 15 s at 94 °C and 30 s at 58 °C on a qTower G3 cycler (Analytic Jena) in sealed qPCR 96-well plates. Primers and probes were used as previously reported^57^. Oligonucleotides (Sequence (5’-3’)): E_Sarbeco_F: ACAGGTACGTTAATAGTTAATAGCGT;

E_Sarbeco_R: ATATTGCAGCAGTACGCACACA;

E_Sarbeco_P1: FAM-ACACTAGCCATCCTTACTGCGCTTCG-BBQ.

### *Mesocricetus auratus* genome annotation

For quantification of gene expression, we used the MesAur 2.0 genome assembly and annotation available via the NCBI genome database (https://www.ncbi.nlm.nih.gov/genome/11998?genome_assembly_id=1585474). The GFF file was converted to GTF using gffread 0.12.7^58^. Where no overlaps were produced, 3’-UTRs in the annotation were extended by 1,000 bp as described previously^59^. Further polishing steps for the GTF file are described on the GitHub page accompanying this paper (https://github.com/Berlin-Hamster-Single-Cell-Consortium/Live-attenuated-vaccine-strategy-confers-superior-mucosal-and-systemic-immunity-to-SARS-CoV-2). The final gtf file used for the analysis is available through GEO (https://www.ncbi.nlm.nih.gov/geo/query/acc.cgi?acc=GSE200596).

### Bulk RNA extraction

To perform RNA bulk sequencing, RNA was isolated from lung tissue using Trizol reagent according to the manufacturer’s instructions (Ambion, Life Technologies). Briefly, 1 ml Trizol was added to the homogenized organ sample and vortexed thoroughly. After an incubation time of 20 min, 200 µl of chloroform were added. The samples were vortexed again and incubated for 10 min at room temperature. Subsequently, tubes were centrifuged at 12,000 × *g* for 15 min at 4 °C and 500 µl of the aqueous phase were transferred into a new tube containing 10 µg GlycoBlue. Isopropanol (500 µl) was added, followed by vortexing, incubating and centrifuging the samples as described above. Thereafter, isopropanol was removed and 1 ml of ethanol (75%) was applied. The tubes were inverted shortly and centrifuged at 8,000 × *g* for 10 min. After freeing the pellet from ethanol, RNA was resuspended in 30 µl of RNase-free water and stored at −80 °C.

### Cell isolation from blood and lungs

White blood cells were isolated from EDTA-blood as previously described; steps included red blood cell lysis and cell filtration before counting. Lung cells (caudal lobe) were isolated as previously described^26,60^; steps included enzymatic digestion, mechanical dissociation and filtration before counting in trypan blue. Buffers contained 2 µg ml^−1^ actinomycin D to prevent de novo transcription during the procedures.

### Cell isolation from nasal cavities

To obtain single-cell suspensions from the nasal mucosa of SARS-CoV-2-challenged hamsters, the skull of each animal was split slightly paramedian so that the nasal septum remained intact on the left side of the nose. The right side of the nose was carefully removed from the cranium and stored in ice-cold 1× PBS with 1% BSA and 2 µg ml^−1^ actinomycin D until further use. Nose parts were transferred into 5 ml Corning Dispase solution supplemented with 750 U ml^−1^ Collagenase CLS II and 1 mg ml^−1^ DNase, and incubated at 37 °C for 15 min. For preparation of cells from the nasal mucosa, the conchae were carefully removed from the nasal cavity and re-incubated in digestion medium for 20 min at 37 °C. Conchae tissue was dissociated by pipetting and pressing through a 70 µm filter with a plunger. Ice-cold PBS with 1% BSA and 2 µg ml^−1^ actinomycin D was added to stop the enzymatic digestion. The cell suspension was centrifuged at 400 × *g* at 4 °C for 15 min and the supernatant discarded. The pelleted nasal cells were resuspended in 5 ml red blood cell lysis buffer and incubated at room temperature for 2 min. Lysis reaction was stopped with 1× PBS with 0.04% BSA and cells centrifuged at 400 × *g* at 4 °C for 10 min. Pelleted cells were resuspended in 1× PBS with 0.04% BSA and 40 µm-filtered. Live cells were counted in trypan blue and viability rates determined using a counting chamber. Cell concentration for scRNA-seq was adjusted by dilution.

### Single-cell RNA sequencing

Isolated cells from blood, lungs and nasal cavities of Syrian hamsters were subjected to scRNA-seq using the 10× Genomics Chromium Single Cell 3’ Gene Expression system with feature barcoding technology for cell multiplexing (details in Supplementary Methods).

### Analysis of single-cell RNA sequencing data

Sequencing reads were initially processed using bcl2fastq 2.20.0 and the multi command of the Cell Ranger 6.0.2 software. For the cellplex demultiplexing, the assignment thresholds were partially adjusted (for details, see the GitHub page at https://github.com/Berlin-Hamster-Single-Cell-Consortium/Live-attenuated-vaccine-strategy-confers-superior-mucosal-and-systemic-immunity-to-SARS-CoV-2). Further processing was done in R 4.0.4 Seurat R 4.0.6 package^61^, as well as R packages ggplot2 3.3.5, dplyr 1.0.7, DESeq2 1.30.1, lme4 1.1–27.1 and dependencies, and in Python 3.9.13 as well as Python packages scanpy 1.9.1, scvelo 0.2.4 and dependencies. In the next step, cells were filtered by a loose quality threshold (minimum of 250 detected genes per cell) and clustered. Cell types were then annotated per cluster and filtered using cell type-specific thresholds (cells below the median or in the lowest quartile within a cell type were removed). The remaining cells were processed using the SCT/integrate workflow^62^ and cell types again annotated on the resulting Seurat object. All code for downstream analysis is available on GitHub at https://github.com/Berlin-Hamster-Single-Cell-Consortium/Live-attenuated-vaccine-strategy-confers-superior-mucosal-and-systemic-immunity-to-SARS-CoV-2.

### Statistics and reproducibility

Details on statistical analysis of sequencing data including pre-processing steps are described in the individual Methods section. Analyses of virological, histopathological, ELISA, cell frequencies and cell number statistics were performed with GraphPad Prism 9. Statistical details are provided in respective figure legends. No statistical method was used to predetermine sample size. Data distribution was assumed to be normal but this was not formally tested. No data were excluded from the analyses. All experiments involving live animals were randomized, other experiments were not randomized. The investigators were blinded to allocation of hamsters during animal experiments and primary outcome assessment (clinical development, virus titrations, qPCR, ELISpot, serology and histopathology). Investigators were not blinded to allocation in other experiments and analyses.

### Reporting summary

Further information on research design is available in the Nature Portfolio Reporting Summary linked to this article.

## Supplementary information

Supplementary Information Supplementary Figs. 1–13, Methods and References.

Reporting Summary

Supplementary Data Statistical source data for supplementary figures.

## Data Availability

Raw sequencing data are available on GEO (https://www.ncbi.nlm.nih.gov/geo/query/acc.cgi?acc=GSE200596), along with bulk RNA-seq read count tables, and h5 matrices and Seurat objects for the scRNA-seq data. Source data are provided with this paper.

## Source data

Source Data Figs. 1–6 and Extended Data Figs. 1–5 Statistical source data.

## Code availability

Code is available on GitHub at https://github.com/Berlin-Hamster-Single-Cell-Consortium/Live-attenuated-vaccine-strategy-confers-superior-mucosal-and-systemic-immunity-to-SARS-CoV-2.
