# Supplementary material for: Live-attenuated vaccine sCPD9 elicits superior mucosal and systemic immunity to SARS-CoV-2 variants in hamsters
Source: Nat Microbiol. 2023 Apr 3;8(5):860–74. doi: 10.1038/s41564-023-01352-8 (PMC10159847; doi:10.1038/s41564-023-01352-8)
Supplement: Supplementary file 1 — Supplementary Figs. 1–13, Methods and References. [file 41564_2023_1352_MOESM1_ESM.pdf]

nature microbiology

Article https://doi.org/10.1038/s41564-023-01352-8

Live-attenuated vaccine sCPD9 elicits superior mucosal and systemic immunity to SARS-CoV-2 variants in hamsters

In the format provided by the authors and unedited

Table of Contents

Supplementary Fig. 1. …………………………………………… page 2

Supplementary Fig. 2………………………………………........page 3

Supplementary Fig. 3. …………………………………………… page 4

Supplementary Fig. 4…………………………………………….. page 5

Supplementary Fig. 5. …………………………………………… page 6

Supplementary Fig. 6…………………………………………….. page 7

Supplementary Fig. 7. …………………………………………… page 8

Supplementary Fig. 8…………………………………………….. page 9

Supplementary Fig. 9. …………………………………………… page 10 – 11

Supplementary Fig. 10…………………………………………… page 12

Supplementary Fig. 11…………………………………………… page 14

Supplementary Fig. 12…………………………………………… page 15

Supplementary Fig. 13…………………………………………… page 16

Supplementary Methods………………………………………… page 17

Supplementary References……..……………………………..page 20

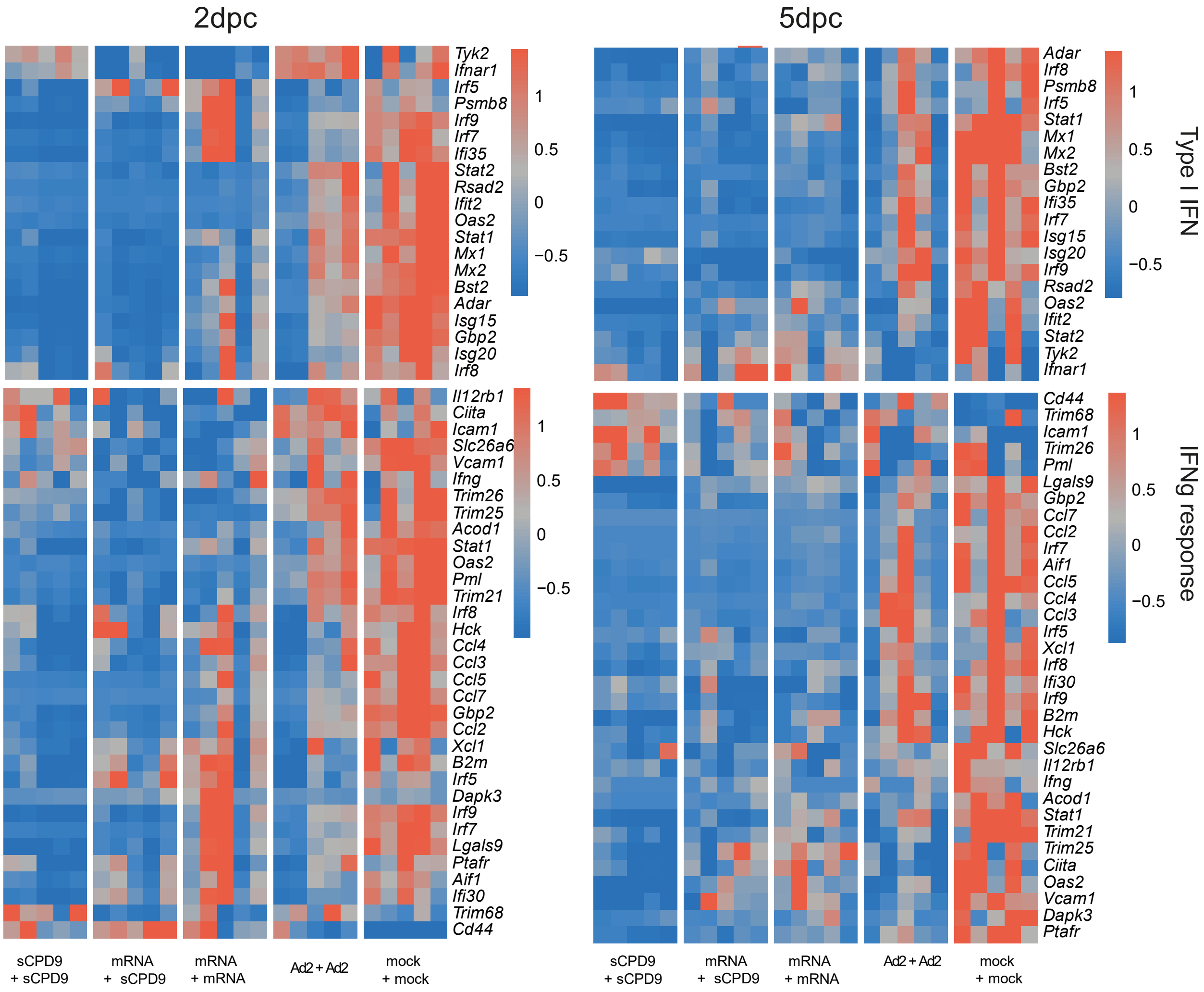

Supplementary Fig. 1. Heatmaps of differently expressed genes involved in the immune response at 2- and 5-days post challenge (dpc) with SARS-CoV-2 were analyzed in the lung tissue of Syrian hamsters after 4 different vaccination strategies using bulk RNA-sequencing. Genes involved in the type I IFN signaling and related to cellular response to IFN-γ were as previously described [1]. Columns represent samples and rows genes. Shown are z-scores of DESeq2-normalized data and color scale ranges from red (10 % upper quantile) to blue (10 % lower quantile), showing up- or down-regulation in expression of the selected genes. N = 5 animals per group.

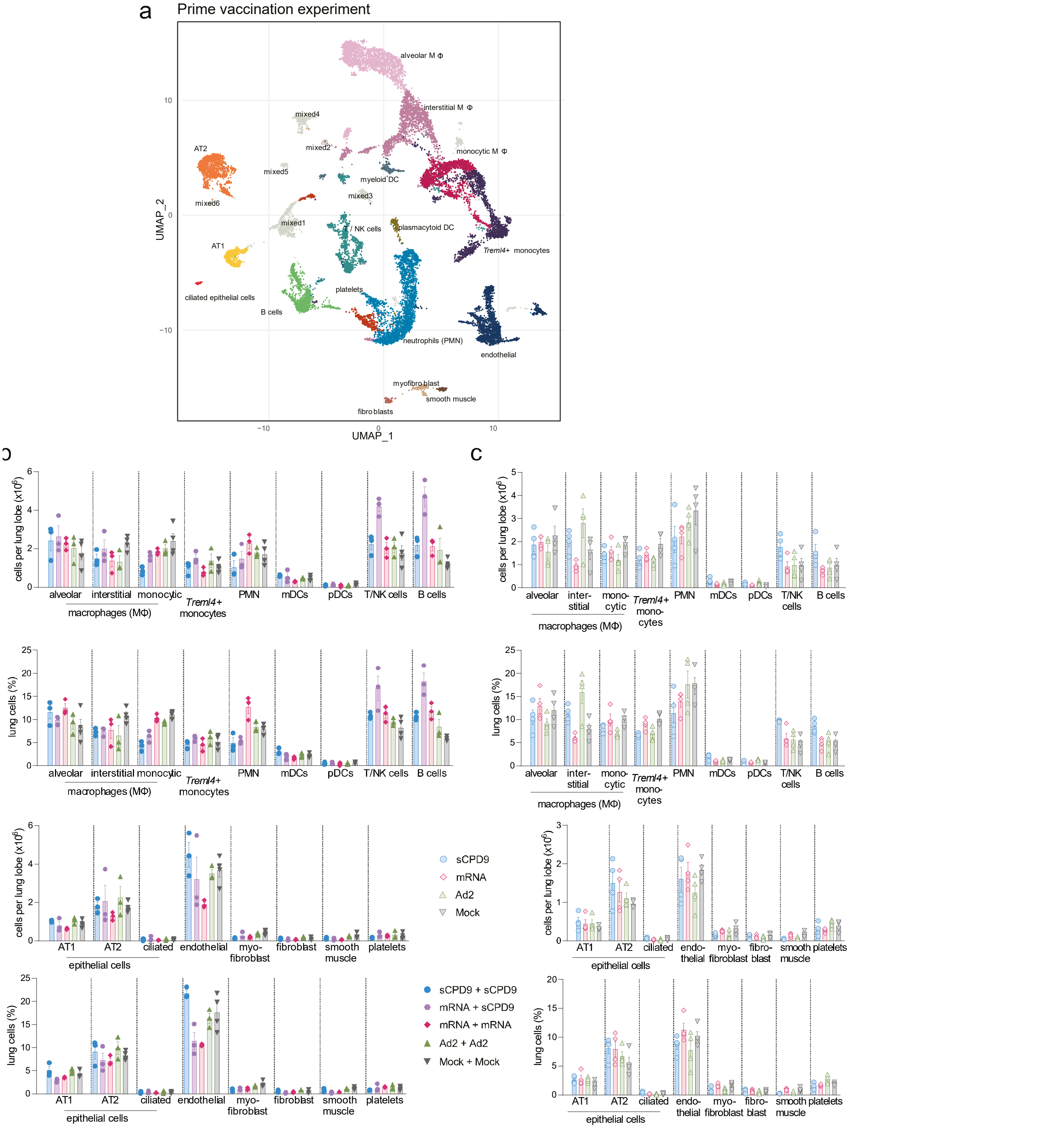

Supplementary Fig. 2. (**a**) Two-dimensional projections of single-cell transcriptomes using UMAP of lung cells from the prime vaccination experiment. Cells are colored by cell types as annotated based on known marker genes. (**b** – **c**) Numbers and percentage of cellular components per lung lobe for (**b**) prime-boost experiment and (**c**) prime-only vaccination experiment. Numbers and frequencies of PMN and monocytic MΦ from Fig. S2b are also shown in Fig. 2c and from Fig. S2c in Fig. 2d. Mean ± SEM. Symbols represent individual hamsters. N_{sCPD9} = 4, n_{mRNA} = 4, n_{Ad2} = 4, n_{mock} = 4, $n_{sCPD9 + sCPD9}$ = 3, $n_{mRNA + sCPD9}$ = 3, $n_{mRNA + mRNA}$ = 3, $n_{Ad2 + Ad2}$ = 3, $n_{mock + mock}$ = 4 individual hamsters as symbols. Prime and prime-boost experiment were performed independently.

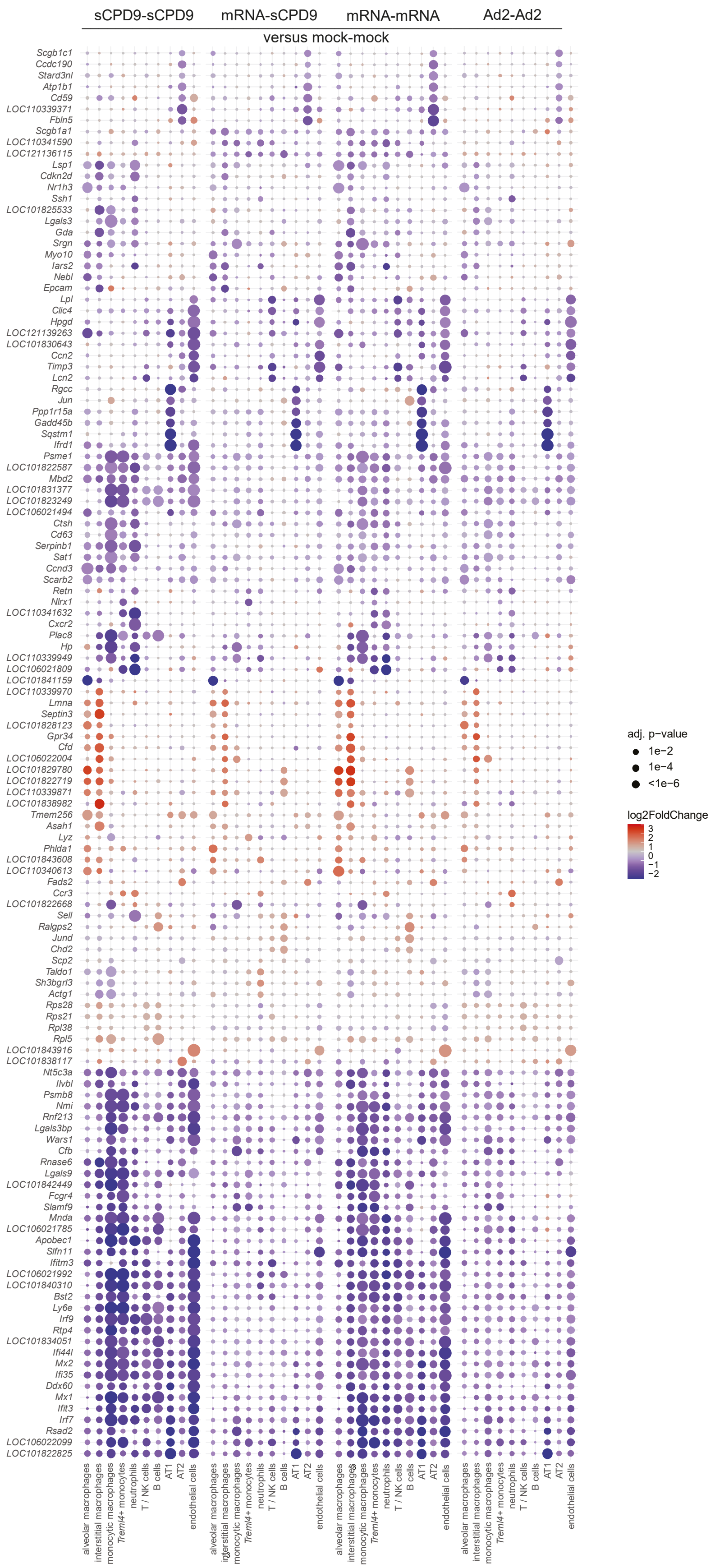

Supplementary Fig. 3. Dotplot showing fold changes of gene expression in indicated cell types of prime-boost vaccinated compared to mock-mock vaccinated animals. Coloration and point size indicate log2-transformed fold changes (FC) and p-values, respectively, in vaccinated compared to mock-mock vaccinated animals. Adjusted (adj) p-values were calculated by DEseq2 using Benjamini–Hochberg corrections of two-sided Wald test p-values. Genes are ordered by unsupervised clustering.

a

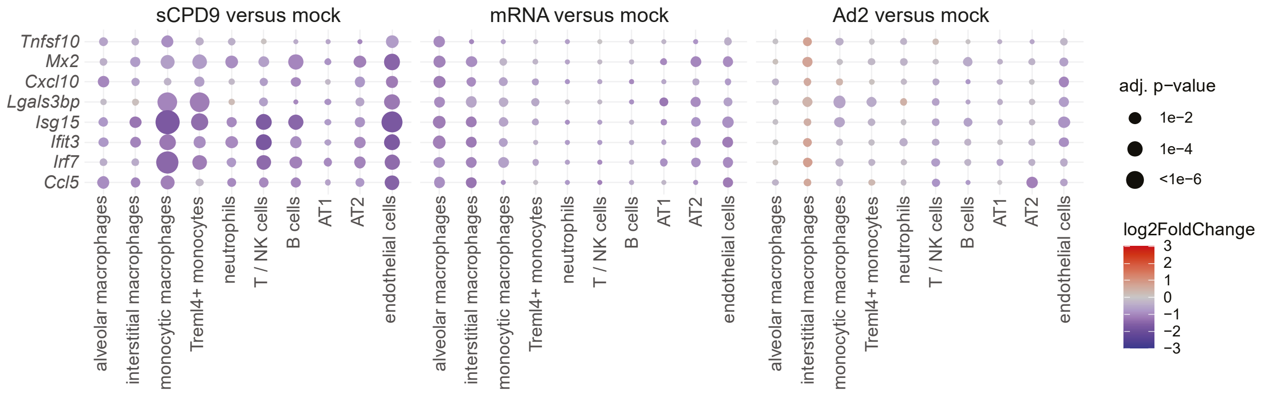

b

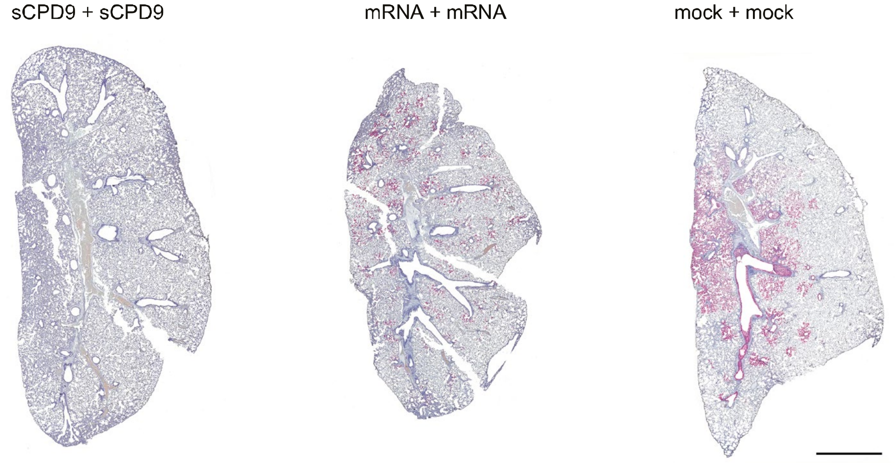

Supplementary Fig. 4. (**a**) Dotplots showing fold changes of gene expression in indicated cell types of the three prime vaccinations compared to mock vaccinated animals. Selected interferon-stimulated genes and pro-inflammatory cytokines are visualized as following. Coloration and point size indicate log2-transformed fold changes (FC) and p-values, respectively, in vaccinated compared to mock-mock vaccinated animals. Adjusted (adj) p-values were calculated by DEseq2 using Benjamini–Hochberg corrections of two-sided Wald test p-values. Genes are ordered by unsupervised clustering. (**b**) Localization of viral RNA (ribonucleic acid) by in situ-hybridization in scanned sections of left lung lobes at 2 dpc in indicated vaccination groups. Red signals: viral RNA, blue: hemalum counterstain. Scale bar represents 3 mm. (**c**) SARS-specific IgG levels against spike in sera from prime only and prime-boost vaccinated hamsters on day 0 and 2 after challenge. Results are shown in optical density (OD) determined at 450nm. Centre (median), Box plot (25th to 75th percentile), with whiskers: (Min to Max) and symbols indicating individual values. Two-way ANOVA and Tukey's multiple comparison test between corresponding prime and prime-boost vaccination regimes. ** $p < 0.01$, and **** $p < 0.0001$. N = 5 individual hamsters per group.

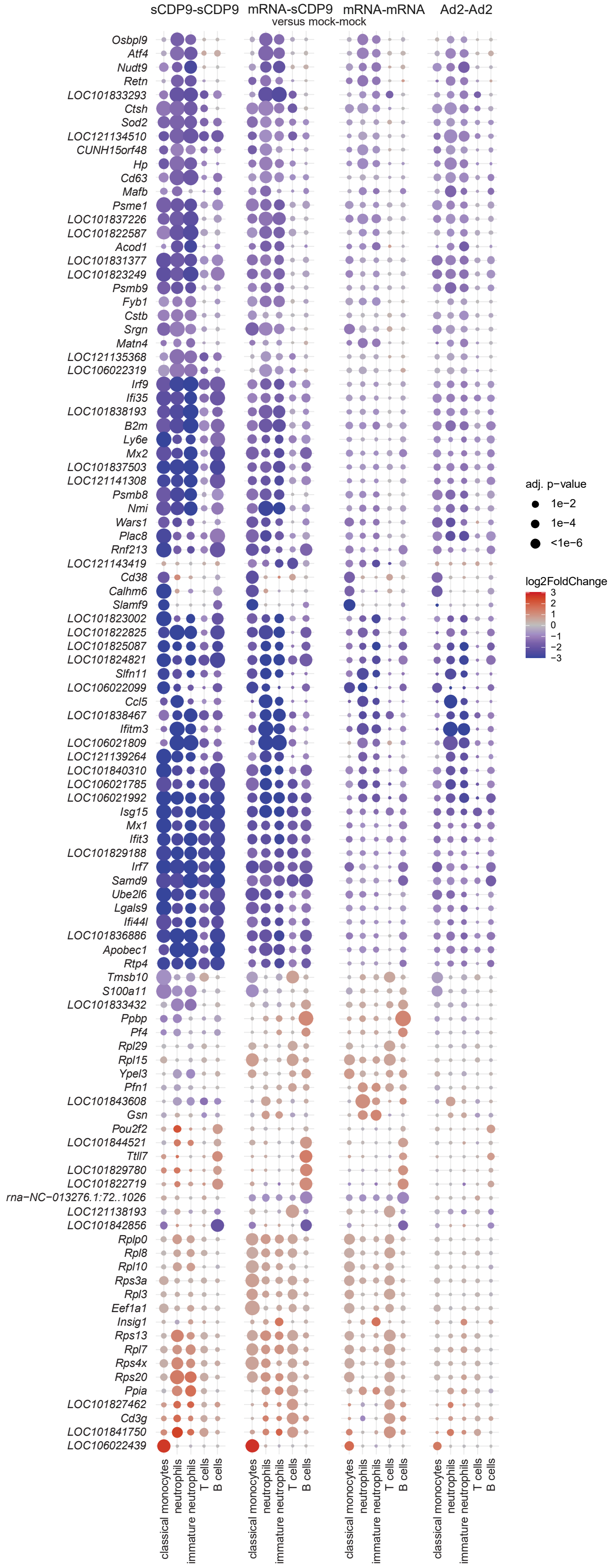

Supplementary Fig. 5. Dotplots showing fold changes of gene expression in indicated cell types of prime-boost vaccinated compared to mock-mock vaccinated animals. Coloration and point size indicate log2-transformed fold changes (FC) and p-values, respectively, in vaccinated compared to mock-mock vaccinated animals. Adjusted (adj) p-values were calculated by DEseq2 using Benjamini–Hochberg corrections of two-sided Wald test p-values. Genes are ordered by unsupervised clustering.

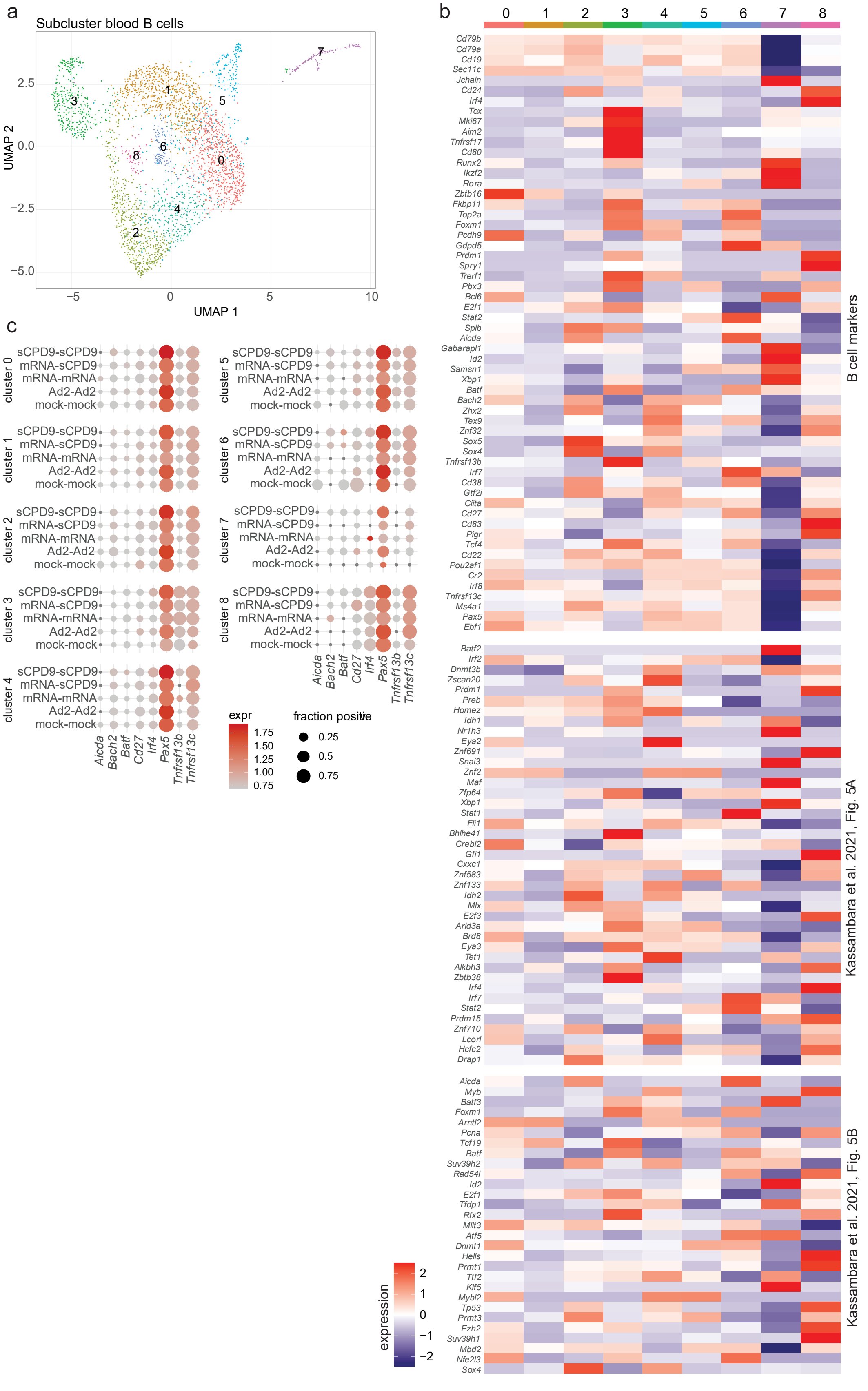

Supplementary Fig. 6. (**a**) Two-dimensional projections of single-cell transcriptomes using UMAP of blood B cells from prime-boost experiment. Cells are colored by clusters. Cluster 7 also contains cells expressing the T cell marker *Cd3e*, and therefore likely represents doublets or artefacts, and was therefore not considered further. (**b**) Heatmaps of classical B cell markers and genes from indicated publications. (**c**) Dotplots showing expression of selected marker genes in blood derived from B cell subcluster analysis. The size of the dot represents the fraction of cells in which at least one UMI of the respective was detected, the color is proportional to the average expression in those cells

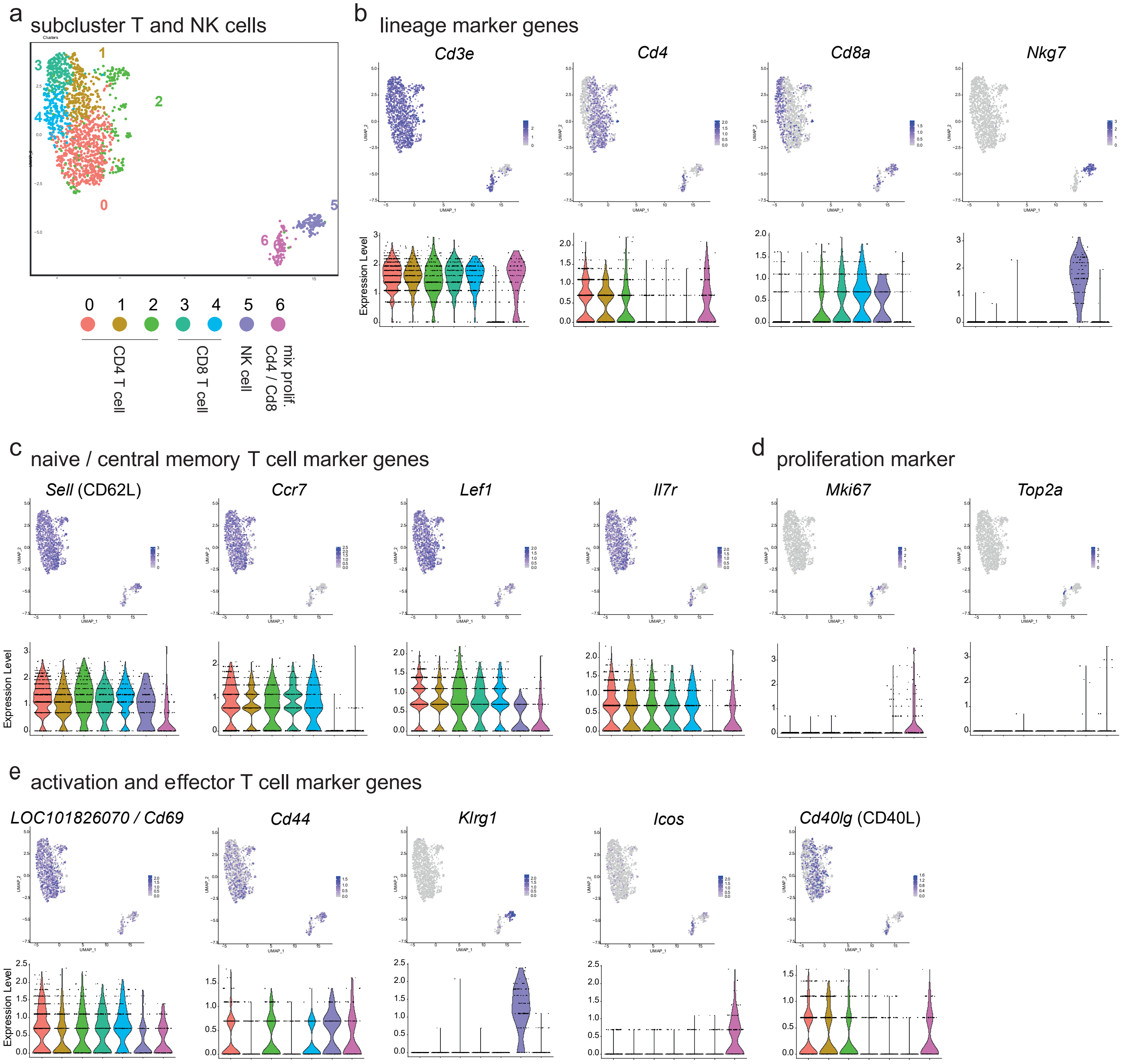

Supplementary Fig. 7. (**a**) Uniform manifold approximation and projection (UMAP) plot of blood T and NK cell subclustering from prime-boost experiment. Colors representing depicted cell types. (**b** – **e**) Feature plots showing gene expression patterns for individual cell markers and violin plots showing expression distributions in each cluster on single-cell level. Each dot represents a single cell. (**b**) Lineage marker genes: *Cd3e*, *Cd4*, *Cd8a*, *Nkg7*. (**c**) Naive / central memory T cell marker genes: *Sell* (CD62L), *Ccr7*, *Lef1*, *Il7r*. (**d**) Proliferation marker genes: *Mki67*, *Top2a*. (**e**) Activation and effector T cell marker genes: *LOC101826070* (*Cd69*), *Cd44*, *Klrg1*, *Icos*, *Cd40lg* (CD40L).

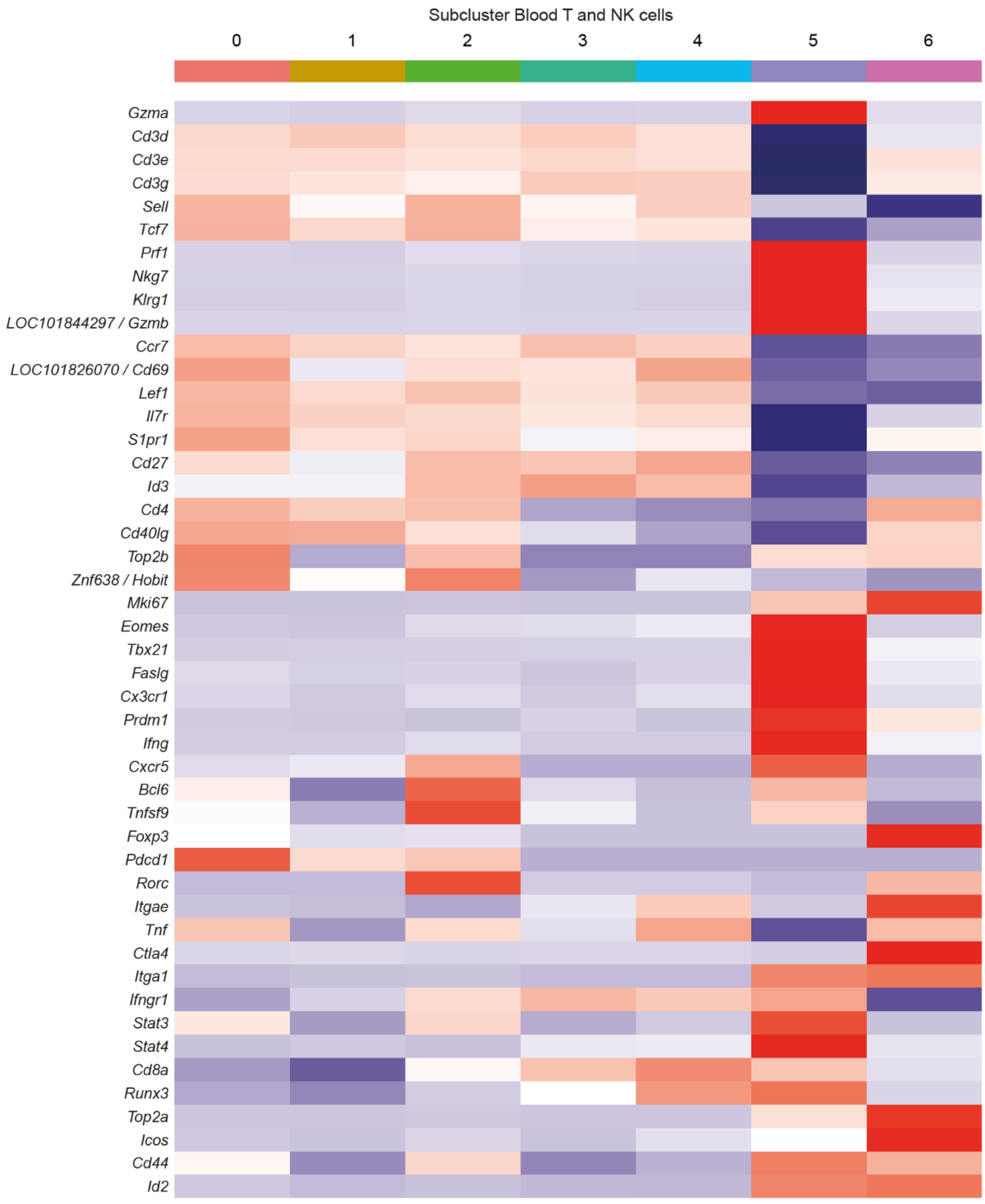

Supplementary Fig. 8. Heatmap of cell marker expression in identified clusters for blood T and NK cell subtypes from prime-boost experiment. Normalized average gene expression levels for cells in a cluster are indicated by coloration: low expression is shown in blue, high expression in red.

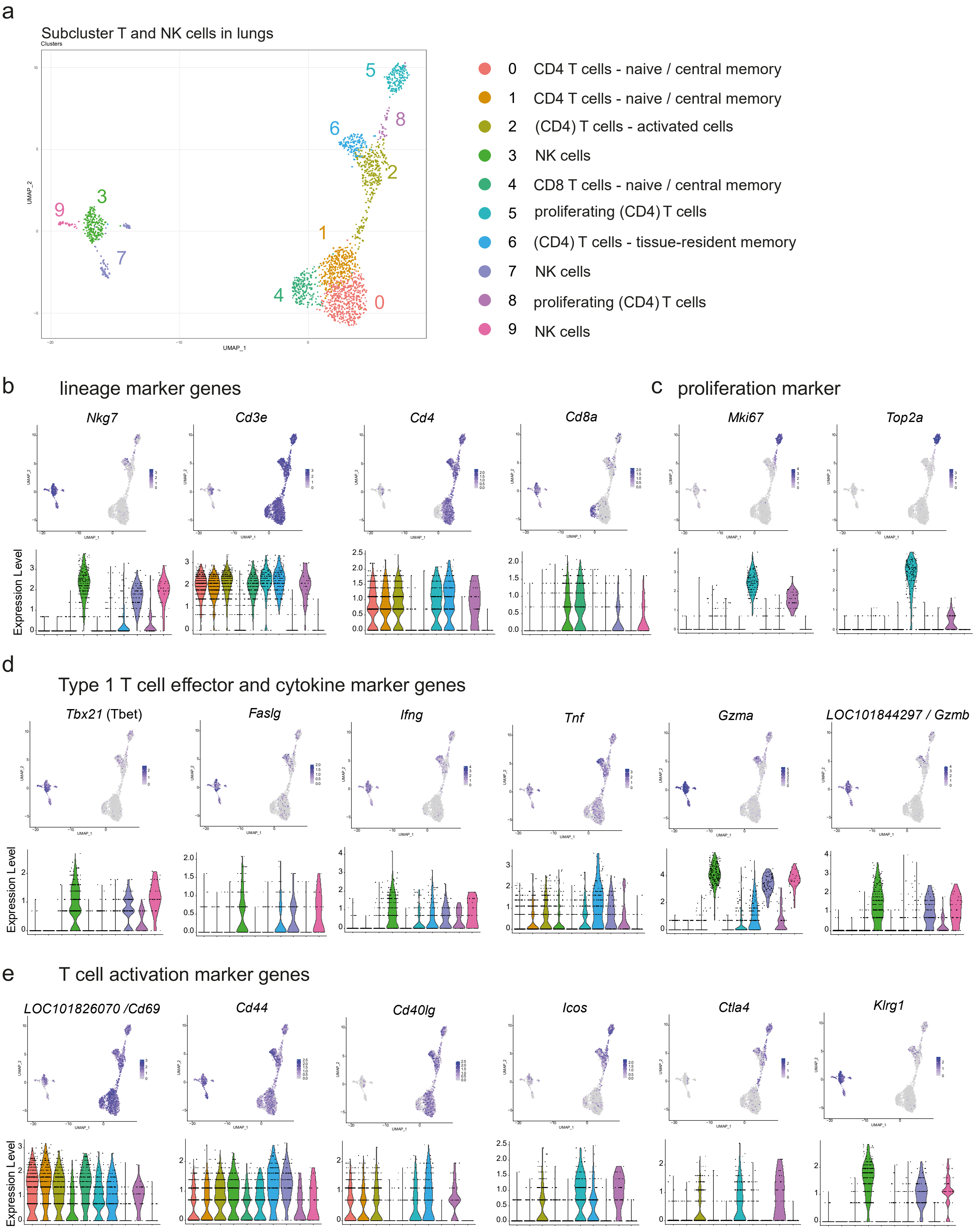
a
Subcluster T and NK cells in lungs
Clusters
0 CD4 T cells - naive / central memory
1 CD4 T cells - naive / central memory
2 (CD4) T cells - activated cells
3 NK cells
4 CD8 T cells - naive / central memory
5 proliferating (CD4) T cells
6 (CD4) T cells - tissue-resident memory
7 NK cells
8 proliferating (CD4) T cells
9 NK cells
UMAP_1
UMAP_2
b lineage marker genes
Nkg7
Cd3e
Cd4
Cd8a
c proliferation marker
Mki67
Top2a
Expression Level
d
Type 1 T cell effector and cytokine marker genes
Tbx21 (Tbet)
Faslg
Ifng
Tnf
Gzma
LOC101844297 / Gzmb
e T cell activation marker genes
LOC101826070 /Cd69
Cd44
Cd40lg
Icos
Ctla4
Klrg1

Supplementary Fig. 9. (**a**) Uniform manifold approximation and projection (UMAP) plot of lung T and NK cell sub-clustering from prime-boost experiment. Colours representing depicted cell types. (**b** – **e**) Feature plots showing gene expression patterns for individual cell markers and violin plots showing expression distributions in each cluster on single-cell level. Each dot represents a single cell. (**b**) Lineage marker genes: *Nkg7*, *Cd3e*, *Cd4*, *Cd8a*. (**c**) Proliferation marker genes: *Mki67*, *Top2a*. (**d**) Type 1 T cell effector and cytokine marker genes: *Tbx21* (Tbet), *Faslg*, *Ifng*, *Tnf*. (**e**) T cell activation marker genes: *LOC101826070* (*Cd69*), *Cd44*, *Cd40lg* (CD40L), *Icos*, *Ctla4*, *Klrg1*.

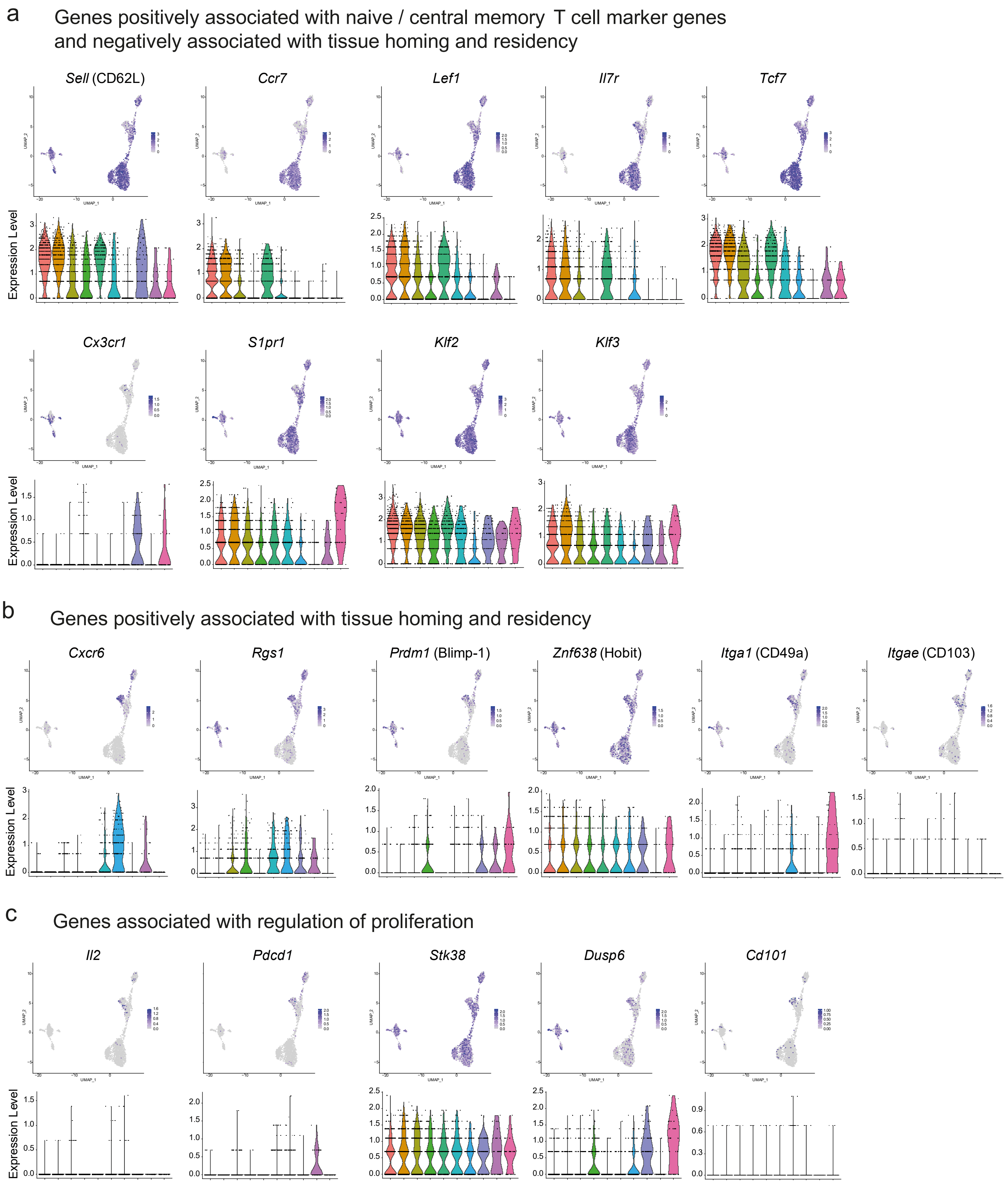

Supplementary Fig. 10. (**a** – **c**) Feature plots showing gene expression patterns for individual cell markers and violin plots showing expression distributions in each cluster on single-cell level from prime-boost experiment. Each dot represents a single cell. (**a**) Genes positively associated with naive / central memory T cell marker genes and negatively associated with tissue homing and residency: *Sell* (CD62L), *Ccr7*, *Lef1*, *Il7r*, *Tcf7*, *Cx3cr1*, *S1pr1*, *Klf2*, *Klf3*. (**b**) Genes

positively associated with tissue homing and residency: *Cxcr6*, *Rgs1*, *Prdm1* (Blimp-1), *Znf638* (Hobit), *Itga1* (CD49a), *Itgae* (CD103). (**c**) Genes associated with regulation of proliferation: *Il2*, *Pdcd1*, *Stk38*, *Dusp6*, *Cd101*.

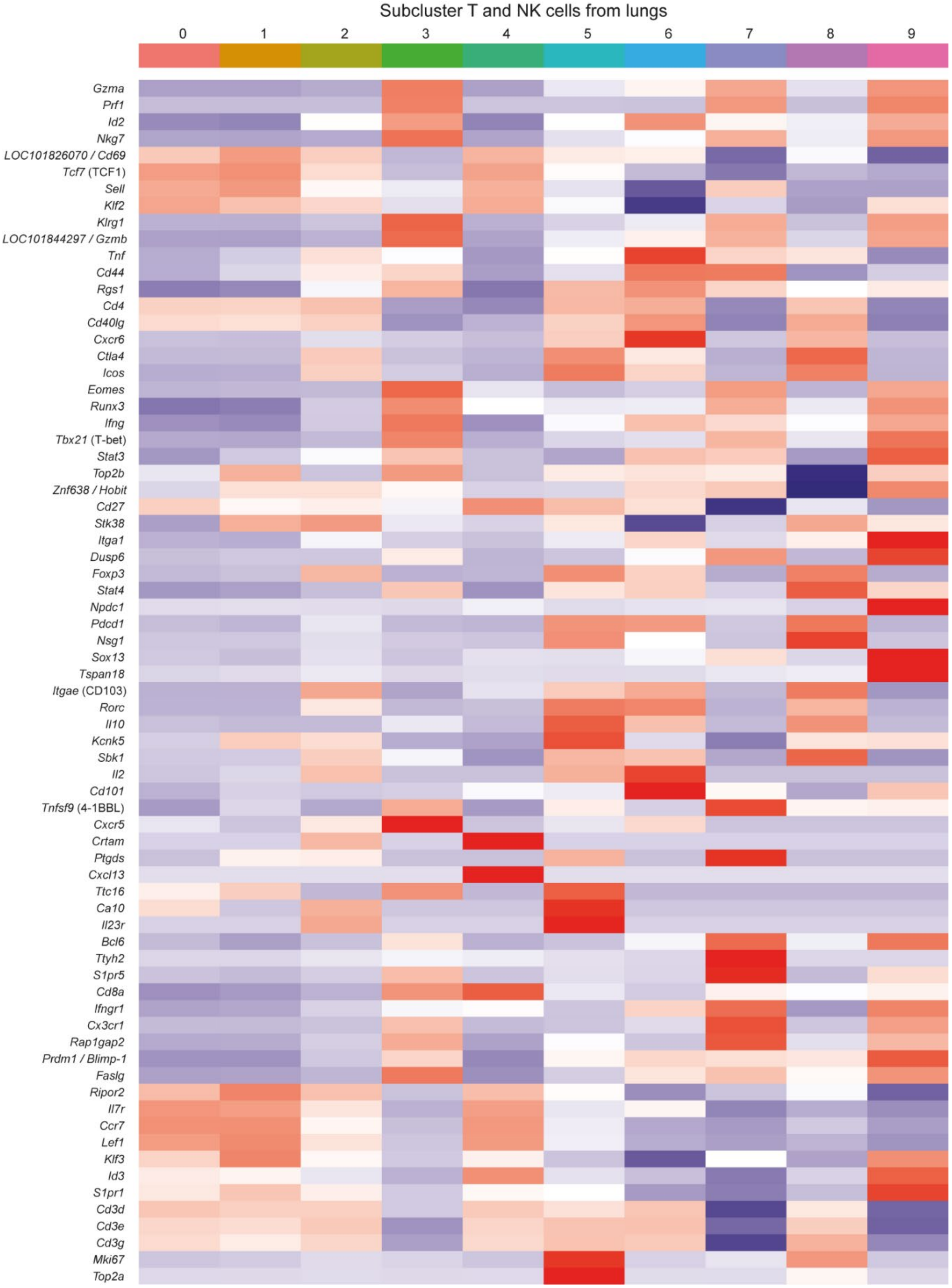

Supplementary Fig. 11. Heatmap of cell marker expression in identified clusters for lung T and NK cell subtypes from prime-boost experiment. Normalized average gene expression levels for cells in a cluster are indicated by coloration: low expression is shown in blue, high expression in red.

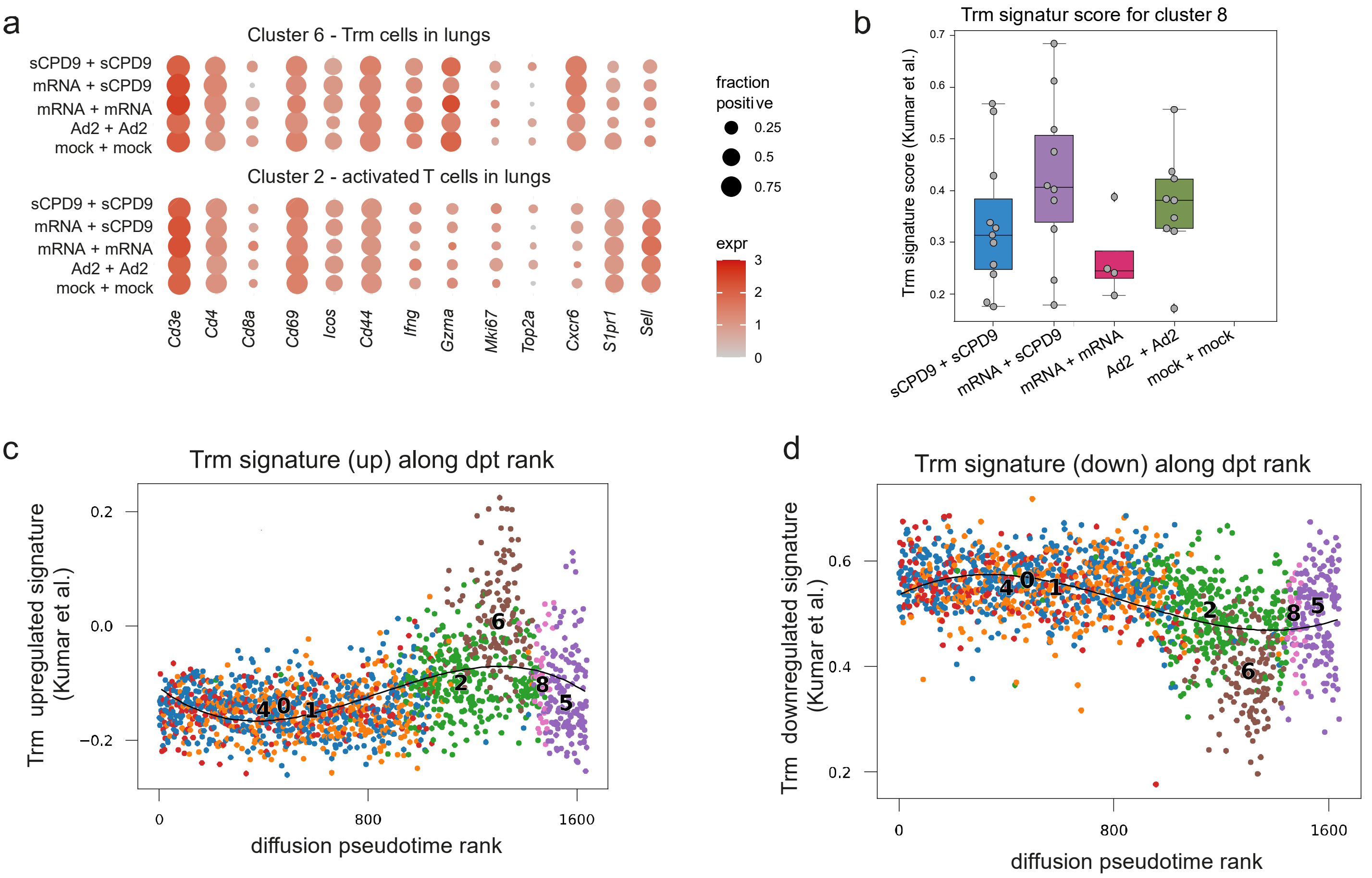

Supplementary Fig. 12. (**a** – **d**) Analysis of T cell subsets by scRNA-seq at 2 dpc in blood and lungs of prime-boost vaccinated hamsters. (**a**), Dotplots showing expression of selected T cell marker genes in the lungs in cluster 6 and 2 derived from T and NK subcluster analysis in Fig. S12A. The size of the dot represents the fraction of cells in which at least one UMI of the respective was detected, the color is proportional to the average expression in those cells. (**b**) Trm signature score for individual cells in cluster 8 according to prime-boost vaccination regimen. Centre (Median), Box (25th to 75th percentile) and whiskers (Min to Max) with individual cells displayed. Circles indicate individual analyzed cells in cluster 8 pooled from $n_{sCPD9 + sCPD9}$ = 3, $n_{mRNA + sCPD9}$ = 3, $n_{mRNA + mRNA}$ = 3, $n_{Ad2 + Ad2}$ = 3, $n_{mock + mock}$ = 4 animals per indicated group (**c**) Trm upregulated signature score as a function of diffusion pseudotime rank with black line showing a polynomial fit of degree three. (**d**) Trm downregulated signature score as a function of diffusion pseudotime rank with black line showing a polynomial fit of degree three.

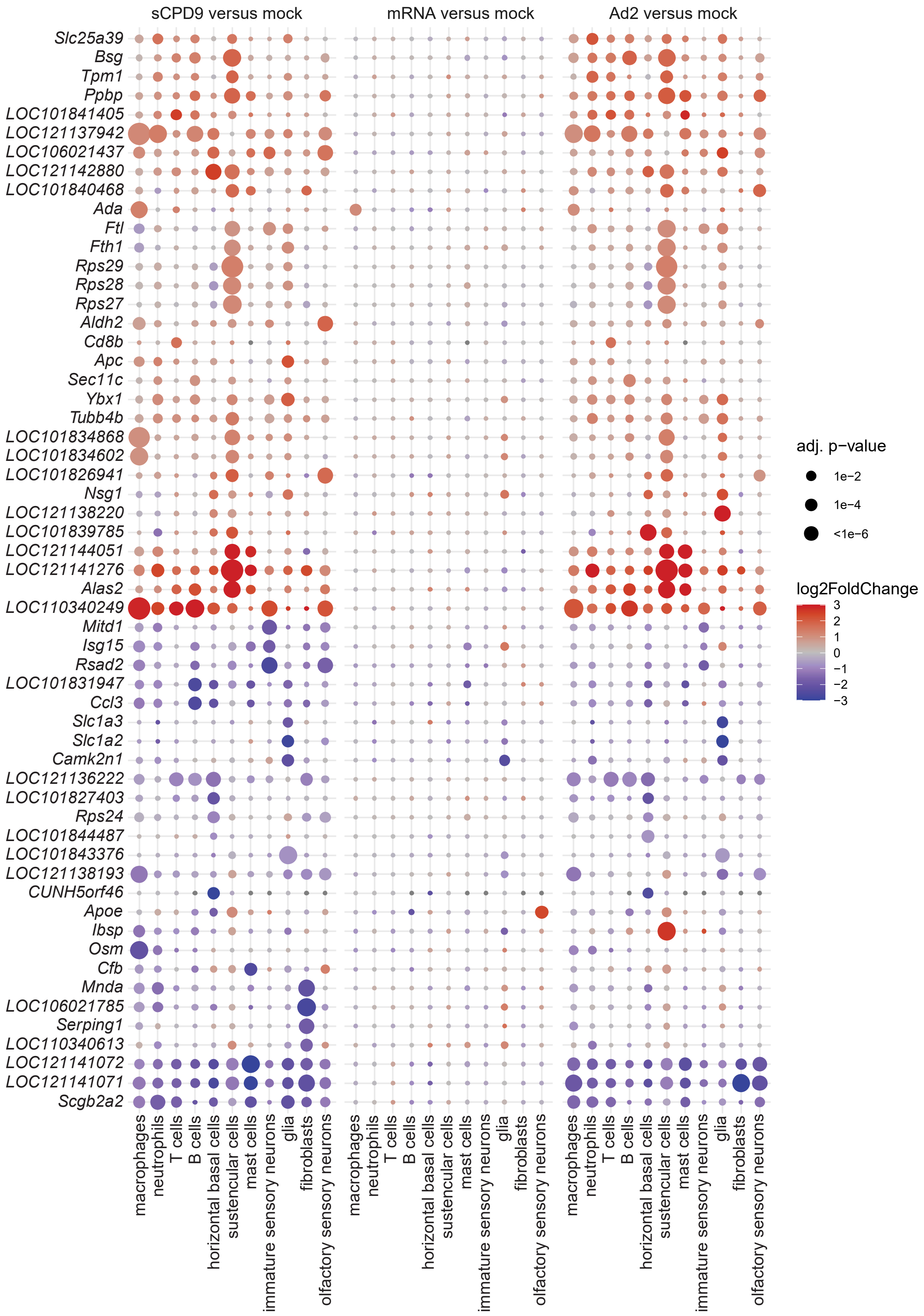

Supplementary Fig. 13. Dotplots showing fold changes of gene expression in indicated cell types of prime vaccinated compared to mock vaccinated animals. Coloration and point size indicate log2-transformed fold changes (FC) and p-

values, respectively, in vaccinated compared to mock vaccinated animals. Adjusted (adj) p-values were calculated by DEseq2 using Benjamini–Hochberg corrections of two-sided Wald test p-values. Genes are ordered by unsupervised clustering.

Supplementary Methods:

Histopathology, immunohistochemistry and in situ-hybridization

Method details: Paraffin-embedded tissues were cut (2 µm thickness), mounted on adhesive glass slides, dewaxed in xylene, rehydrated in descending grades of alcohol, and endogenous peroxidase was inhibited. Antigen retrieval was performed using microwave heating (600 W) in 10 mM citric acid (pH 6.0) for 12 min for SARS-CoV-1 nucleoprotein antibody (Sino Biological Inc.; Beijing, China) and using recombinant protease from Streptomyces griseus (PanReac AppliChem, Darmstadt, Germany) for 13 min at 37°C for IgA antibody. For blockage of non-specific antibody binding incubation with 8 % Roti-Immunoblock (Roth, Karlsruhe, Germany) and 20 % normal goat serum for 30 min was implemented. Anti-SARS-CoV-1 NP mouse monoclonal antibody (Sino Biological Inc.; Beijing, China, dilution: 1:500) and rabbit anti hamster IgA antibody (Brookwood Biomedical, Jemison, AL, dilution: 1:250) were incubated at 4°C overnight followed by washing and incubation with a secondary biotinylated goat anti-mouse IgG antibody (dilution: 1:200, Vector Laboratories, Burlingame, California, USA). For color development freshly prepared avidin-biotin-peroxidase complex (ABC) solution (Vectastain Elite ABC Kit; Vector Laboratories) was incubated for 30 min followed by incubation with diaminobenzidine tetrahydrochloride (Merck, Darmstadt, Germany) for 4 min. Slides were counterstained with Mayer’s hematoxylin.

An Olympus BX41 microscope with a DP80 Microscope Digital Camera and the cellSensTM Imaging Software, Version 1.18 (Olympus Corporation, Münster, Germany) was utilized for histopathological evaluations and photographs. Slides were automatically digitized with the Aperio CS2 slide scanner (Leica Biosystems Imaging Inc., Vista, CA, USA) and overviews were generated by using the image Scope Software (Leica Biosystems Imaging Inc.).

SARS-specific Ig measurement by Enzyme-linked Immunosorbent Assays (ELISA) from serum and nasal washes

Detailed experimental procedure: MEDISORP plates (Thermofisher, MW96F straight) were used to conduct this assay and coated in two steps. First, SARS-CoV-2 spike protein (Acro Biosystems, SARS-CoV-2 S protein (D614G), His Tag, Super stable trimer) was diluted in 1× PBS to a final concentration of 20 µg/mL, and 5 µL of the respective dilution were applied to each well. For serum samples, additional plates were coated with SARS-CoV-2 nucleocapsid protein (Ray Biotech, (QHD43423) recombinant nucleocapsid protein) and SARS-CoV-2 ORF3a protein (Invitrogen SARS-CoV-2 ORF3a (aa126-275), His Tag Recombinant Protein) as described above. All antigens were diluted in tubes with a low binding capacity for proteins (Eppendorf Protein LoBind Tube 1.5 ml). Second, 45 µL of coating buffer (diH2O + 5.3 g Na2CO3 (50 mM) + anhydrous 4.2 g NaHCO3 (50 mM), pH 9.6) were added per well, followed by an incubation time of 12 h at 4°C. Subsequently, the plates were washed four times with washing buffer (1 × PBS + 0.05 % Tween20) and blocked with 1× PBS + 1 % BSA + 10 % FCS for 1 h. Serum and nasal washes (each sample in duplicate) were diluted 1:100 in dilution buffer (1 × PBS + 2 % BSA + 0.1 % Tween 20) and 50 µL were pipetted to each well. The plates were covered and incubated for 2 h at room temperature before the washing step was repeated. Thereafter, 50 µL of secondary antibody diluted 1:1000 was applied per well. For nasal washes, an HRP-conjugated Rabbit Anti-Hamster IgA (Brookwood biomedical) was used, whilst an HRP-conjugated Goat anti-Hamster IgG (Invitrogen, Invitrogen Goat anti-Hamster IgG(H+L) Cross-Adsorbed, Secondary Antibody, HRP) was administered to detect IgG levels in serum samples. The covered plates were incubated for 1 h at room temperature and washed again as described above. Subsequently, 50 µL of 3,3',5,5'-Tetramethylbenzidine (TMB) were added followed by another 15 min of incubation. After applying 50 µL stop solution (1 M H2SO4) per well, SpectraMax Plus384 was used to read the plates at 450 nm and 570 nm to assess optical density (OD).

Single-cell RNA sequencing

In the prime only experiment, ~ 500,000 cells per blood sample, 1,000,000 cells per lung sample, and 1,000,000 per nasal cavity sample underwent cell multiplex oligo

(CMO) labeling according to manufacturers' instructions (3' CellPlex Kit Set A; 10× Genomics), using the classical labelling protocol. In the prime-boost experiment, ~150,000 cells per blood sample, ~350,000 cells per lung sample, and ~350,000 per nasal cavity sample underwent cell multiplex oligo (CMO) labeling according to manufacturers' instructions (3' CellPlex Kit Set A; 10× Genomics), using the 96-well plate labelling protocol for low cell numbers to minimize protocol duration and cell manipulation (https://assets.ctfassets.net/an68im79xiti/4G3MABhAGLG8oAEyrSiz7L/a6e4f84663 4a50f0d44025bab4b0f858/CG000426_PlateBasedSamplePrep_RevC.pdf.). In the first two experimental runs (prime experiment), pools were generated from 12 samples derived from all three organ types. De-multiplexing difficulties for blood samples from mixed pools led to a change in protocol for the third and fourth experimental run (prime-boost experiment). Here, multiplex pools were generated from 8 samples of the same organ origin only.

Oligo labelled cells were pooled accordingly without prior counting to minimize protocol duration, assuming equal cell loss between samples during the labelling procedures. Pooled cells were 40-µm filtered, counted and cell concentration adjusted to 1450 -1,600 cells /µL, resulting in loading of 49,500 pooled cells per lane aiming to recover 30,000 cells. Pools consisting of 12 samples were loaded onto 4 Chromium Next GEM Chip G lanes, pools consisting of 8 samples were loaded onto 2 or 3 lanes (blood samples on 2 lanes, lung and nasal cavity on 3 lanes). Loaded cells underwent partitioning, barcoding and mRNA reverse transcription in Gel-Beads-in-Emulsions following the instructions of Chromium Next GEM Single Cell 3' Reagent Kits v.3.1 (Dual Index) provided by the manufacturer (10× Genomics). Viability rates of nasal cavity cells did not allow for qualitative results for the prime-boost experiment. Library sequencing was performed on a Novaseq 6000 device (Illumina), with SP4 flow cells (read1: 28 nt; read2: 150 nt).

Supplementary References:

1. Winkler, E.S. et al. SARS-CoV-2 infection of human ACE2-transgenic mice causes severe lung inflammation and impaired function. *Nat Immunol* 21, 1327-1335 (2020).
