# Supplementary material for: Live-attenuated vaccine sCPD9 elicits superior mucosal and systemic immunity to SARS-CoV-2 variants in hamsters
Source: Nat Microbiol. 2023 Apr 3;8(5):860–74. doi: 10.1038/s41564-023-01352-8 (PMC10159847; doi:10.1038/s41564-023-01352-8)
Supplement: Supplementary file 2 — Reporting Summary [file 41564_2023_1352_MOESM2_ESM.pdf]

nature portfolio

Corresponding author(s): Jakob Trimpert

Last updated by author(s): Feb 24, 2023

Reporting Summary

Nature Portfolio wishes to improve the reproducibility of the work that we publish. This form provides structure for consistency and transparency in reporting. For further information on Nature Portfolio policies, see our Editorial Policies and the Editorial Policy Checklist.

Statistics

For all statistical analyses, confirm that the following items are present in the figure legend, table legend, main text, or Methods section.

n/a	Confirmed	
☐	☒	The exact sample size (*n*) for each experimental group/condition, given as a discrete number and unit of measurement
☐	☒	A statement on whether measurements were taken from distinct samples or whether the same sample was measured repeatedly
☐	☒	The statistical test(s) used AND whether they are one- or two-sided<br>*Only common tests should be described solely by name; describe more complex techniques in the Methods section.*
☐	☒	A description of all covariates tested
☐	☒	A description of any assumptions or corrections, such as tests of normality and adjustment for multiple comparisons
☐	☒	A full description of the statistical parameters including central tendency (e.g. means) or other basic estimates (e.g. regression coefficient) AND variation (e.g. standard deviation) or associated estimates of uncertainty (e.g. confidence intervals)
☐	☒	For null hypothesis testing, the test statistic (e.g. *F*, *t*, *r*) with confidence intervals, effect sizes, degrees of freedom and *P* value noted<br>*Give P values as exact values whenever suitable.*
☒	☐	For Bayesian analysis, information on the choice of priors and Markov chain Monte Carlo settings
☒	☐	For hierarchical and complex designs, identification of the appropriate level for tests and full reporting of outcomes
☒	☐	Estimates of effect sizes (e.g. Cohen's *d*, Pearson's *r*), indicating how they were calculated

Our web collection on statistics for biologists contains articles on many of the points above.

Software and code

Policy information about availability of computer code

Data collection	cellSens 1.18, Image Scope 12.3, bcl2fastq 2.20.0, cellranger 6.0.2
Data analysis	gffread 0.12.7, Prism 9, Python 3.9.13, and R 4.0.4. Python packages used: scanpy 1.9.1, scvelo 0.2.4, and dependencies. R packages used: ggplot2 3.3.5, Seurat 4.0.6, dplyr 1.0.7, DESeq2 1.30.1, lme4 1.1-27.1, and dependencies. Code is available through GitHub at https://github.com/Berlin-Hamster-Single-Cell-Consortium/Live-attenuated-vaccine-strategy-confers-superior-mucosal-and-systemic-immunity-to-SARS-CoV-2.

For manuscripts utilizing custom algorithms or software that are central to the research but not yet described in published literature, software must be made available to editors and reviewers. We strongly encourage code deposition in a community repository (e.g. GitHub). See the Nature Portfolio guidelines for submitting code & software for further information.

Data

Policy information about availability of data

All manuscripts must include a data availability statement. This statement should provide the following information, where applicable:

- Accession codes, unique identifiers, or web links for publicly available datasets
- A description of any restrictions on data availability
- For clinical datasets or third party data, please ensure that the statement adheres to our policy

Raw sequencing data is available on GEO (https://www.ncbi.nlm.nih.gov/geo/query/acc.cgi?acc=GSE200596), along with bulk RNA-seq read count tables, and h5 matrices and Seurat objects for the scRNA-seq data.

Human research participants

Policy information about studies involving human research participants and Sex and Gender in Research.

Reporting on sex and gender	N/A
Population characteristics	N/A
Recruitment	N/A
Ethics oversight	N/A

Note that full information on the approval of the study protocol must also be provided in the manuscript.

Field-specific reporting

Please select the one below that is the best fit for your research. If you are not sure, read the appropriate sections before making your selection.

☒ Life sciences ☐ Behavioural & social sciences ☐ Ecological, evolutionary & environmental sciences

For a reference copy of the document with all sections, see nature.com/documents/nr-reporting-summary-flat.pdf

Life sciences study design

All studies must disclose on these points even when the disclosure is negative.

Sample size	No statistical method was used to predetermine sample size. Instead, we selected a sample size of n = 3-10 animals per time point and treatment based on our previous experience with SARS-CoV-2 vaccination and infection of Syrian hamsters. To adhere to the 3R principle, we reduced the number of animals used in this study to the minimum that had been experimentally determined in our previous studies (DOI: 10.1016/j.celrep.2021.109493 and DOI: 10.1126/sciadv.abk0172). We also referred to examples from other authors (DOI: 10.1038/s41467-021-26178-y and DOI: https://doi.org/10.1038/s41598-022-15238-y). These publications demonstrate the suitability of the chosen sample size and provide evidence that the number of animals used was the minimum required for the study.
Data exclusions	We aimed to include all relevant data that was generated during the study. However, in some cases, animals were excluded from certain analyses due to a lack of available material. Specifically, for the serum-neutralisation tests and ELISAs, some animals were not included due to a lack of serum. It is important to note that the data for these animals was not excluded, because it was never generated.
Replication	We performed a total of four independent animal experiments to support the findings of this study. These included one experiment with prime-only vaccination, one with prime-boost vaccination, one to determining antigen specificity of cellular responses, and one to study onward transmission of the challenge virus. The outcome of these independent experiments does substantiate our findings.<br>All virological assays (titrations, qPCR, SNT and ELISA) were performed in duplicates (technical replicates) for each animal included in the study. All replication attempts were successful. The respective mean values were then used as individual data points for biological replicates (individual animals). To ensure robust data analysis, we reported data generated from a minimum of 3 biological replicates (individual animals) for all analyses.
Randomization	Hamsters were randomly assigned to groups for all experiments. In other experiments, sample allocation was not random. Experiments were controlled by appropriate controls such as standard curves and positve and negative controls.
Blinding	All animal experimentation was performed blindly. To this end, animals were assigned consecutive numbers, all personal conducting the experiment and subsequent analysis, including histopathology, virological examination, serology, ELISpot, preparation for and sequencing of samples, were only aware of the respective animal number, not of the treatment the animal had received. The person performing vaccinations was aware of the vaccine each hamster received, which is technically necessary due to different handling and application routes. However, this person was different from the personal that monitored and weighed the animals or performed further analyses. In later analyses, such as analysis of sequencing data, the investigators were not blinded. Blinding was not deemed necessary in this case as data had

been already generated at that point.

Reporting for specific materials, systems and methods

We require information from authors about some types of materials, experimental systems and methods used in many studies. Here, indicate whether each material, system or method listed is relevant to your study. If you are not sure if a list item applies to your research, read the appropriate section before selecting a response.

Materials & experimental systems

n/a	Involved in the study
☐	☒ Antibodies
☐	☒ Eukaryotic cell lines
☒	☐ Palaeontology and archaeology
☐	☒ Animals and other organisms
☒	☐ Clinical data
☒	☐ Dual use research of concern

Methods

n/a	Involved in the study
☒	☐ ChIP-seq
☒	☐ Flow cytometry
☒	☐ MRI-based neuroimaging

Antibodies

Antibodies used

ELISA: Rabbit Anti-Hamster IgA, HRP-conjugated (Brookwood Biomedical, Jemison, AL) sab3003a 005002, used at 1:1000 dilution
EILSA: Goat anti-Syrian Hamster IgG (H+L), HRP-conjugated, (Invitrogen, Fisher Scientific, Schwerte, Germany) PA129626, used at 1: 1000 dilution
IHC: Anti-SARS-CoV-1 NP mouse monoclonal antibody, Clone #05, (Sino Biological Inc.; Beijing, China) 40143-MM05, used at 1:500 dilution
IHC: Rabbit Anti-Hamster IgA antibody (Brookwood Biomedical, Jemison, AL) sab3001a, used at 1:250 dilution
IHC: Biotinylated Goat-Anti-Mouse IgG antibody (Vector Laboratories, Burlingame, California USA) BP-9200-50, used at 1:200 dilution

Validation

ELISA: Brookwood Biomedical, sab3003a 005002 was validated on unrelated hamster serum and nasal wash samples in our lab.
Invitrogen PA129626 was validated by the manufacturer: https://www.thermofisher.com/antibody/product/Goat-anti-Syrian-Hamster-IgG-H-L-Secondary-Antibody-Polyclonal/PA1-29626
IHC: Anti-SARS-CoV-1 NP mouse monoclonal antibody, Sino Biological 40143-MM05, validated by the manufacturer used for SARS-CoV-2 IHC by others (DOI: https://doi.org/10.1038/s41467-021-22580-8). The secondary antibody Vector Laboratories BP-9200-50 has been used for detection of mouse IgG by IHC in multiple previous publications (see: https://vectorlabs.com/immpress-hrp-anti-mouse-igg-rat-adsorbed-peroxidase-polymer-detection-kit.html).

Eukaryotic cell lines

Policy information about cell lines and Sex and Gender in Research

Cell line source(s)

Vero E6 ATCC (ATCC CRL-1586)
Vero E6 TMPRSS (NIBSC 100978)
Calu-3 (ATCC HTB-55)
A549 (CCL-185)

Authentication

No authentification was performed prior to experiment start. As part of routine quality checks, Vero E6 cells were authentificated by Deutsche Sammlung von Mikroorganismen und Zellkulturen after experiments were finished. Authentification confirmed the identity of the cells. Cells were used exclusively for SARS-CoV-2 preparation and/or plaque assays.

Mycoplasma contamination

All cell lines tested negative for Mycoplasma spp. prior to the start of experiments.

Commonly misidentified lines
(See ICLAC register)

None

Animals and other research organisms

Policy information about studies involving animals; ARRIVE guidelines recommended for reporting animal research, and Sex and Gender in Research

Laboratory animals

Mesocricetus auratus (Syrian hamster, also known as golden hamster), Janvier labs, Outbred hamster, RjHan:AURA, nine to eleven weeks of age at experiment start.

Wild animals

N/A

Reporting on sex

Female and male hamsters were used and equally distributed in most groups of this study. To enable co-housing, the onward transmission experiment was performed with male animals only.There is no obvious difference between the study outcome for

	males and females, this study however was designed to include both sexes to determine an overall similar or greatly divergent outcome for males and females, it was not designed to identify sex-specific differences in response to vaccination or infection.
Field-collected samples	N/A
Ethics oversight	All animal experiments were performed in compliance with relevant institutional, national, and international guidelines for care and humane use of animal and approved by the Landesamt für Gesundheit und Soziales in Berlin, Germany (permit number 0086/20).

Note that full information on the approval of the study protocol must also be provided in the manuscript.
